# Antibacterial Free Fatty Acids and Monoglycerides: Biological Activities, Experimental Testing, and Therapeutic Applications

**DOI:** 10.3390/ijms19041114

**Published:** 2018-04-08

**Authors:** Bo Kyeong Yoon, Joshua A. Jackman, Elba R. Valle-González, Nam-Joon Cho

**Affiliations:** 1 School of Materials Science and Engineering and Centre for Biomimetic Sensor Science, Nanyang Technological University, 50 Nanyang Drive, Singapore 637553, Singapore; bokyeong001@e.ntu.edu.sg (B.K.Y.); jjackman@ntu.edu.sg or jjackman@stanford.edu (J.A.J.); elbaruth001@e.ntu.edu.sg (E.R.V.-G.); 2 Division of Gastroenterology and Hepatology, Department of Medicine, Stanford University School of Medicine, Stanford, CA 94305, USA

**Keywords:** antimicrobial lipid, fatty acid, monoglyceride, antibacterial, therapy, phospholipid membrane

## Abstract

Antimicrobial lipids such as fatty acids and monoglycerides are promising antibacterial agents that destabilize bacterial cell membranes, causing a wide range of direct and indirect inhibitory effects. The goal of this review is to introduce the latest experimental approaches for characterizing how antimicrobial lipids destabilize phospholipid membranes within the broader scope of introducing current knowledge about the biological activities of antimicrobial lipids, testing strategies, and applications for treating bacterial infections. To this end, a general background on antimicrobial lipids, including structural classification, is provided along with a detailed description of their targeting spectrum and currently understood antibacterial mechanisms. Building on this knowledge, different experimental approaches to characterize antimicrobial lipids are presented, including cell-based biological and model membrane-based biophysical measurement techniques. Particular emphasis is placed on drawing out how biological and biophysical approaches complement one another and can yield mechanistic insights into how the physicochemical properties of antimicrobial lipids influence molecular self-assembly and concentration-dependent interactions with model phospholipid and bacterial cell membranes. Examples of possible therapeutic applications are briefly introduced to highlight the potential significance of antimicrobial lipids for human health and medicine, and to motivate the importance of employing orthogonal measurement strategies to characterize the activity profile of antimicrobial lipids.

## 1. Introduction

Molecular design principles underpin the structure and function of biological assemblies such as cells, and amphiphilic molecules drive the spontaneous self-assembly of key architectural elements like phospholipid membranes [1,2,3]. Understanding the role of molecular self-assembly in directing the formation of biological macromolecular structures, including lipid bilayers, proteins, and assemblies thereof, is part of the nanoarchitectonics field [4,5], and such insights can help solve outstanding biomedical problems. Within this scope, one of the greatest public health problems in the world today is the growing rise of antibiotic-resistant bacteria and the associated challenges to treat and prevent bacterial infections [6]. Less than a century ago, the world’s first antibiotic, penicillin, was discovered, and the specificity of antibiotics to inhibit bacterial enzymes and other proteins necessary for bacterial cell function proved highly effective and a remarkable example of molecular pharmaceutics, as evidenced by marked improvements in healthcare capabilities to treat bacterial infections. As a result, many formerly fatal or debilitating diseases caused by bacterial pathogens were suddenly curable with antibiotic treatment [7].

With high potency and working against a broad spectrum of bacterial targets, antibiotics became the standard drug option to treat bacterial infections, and are also widely used as precautionary measures to treat suspected infections, even when the microbial origin is unknown and could be bacterial, fungal, or viral among other possibilities. Antibiotics are also administered prophylactically in cases where bacterial infections might arise, such as after surgical operations. In addition, antibiotics are commonly used in the agricultural sector to not only treat and prevent bacterial infections among livestock, but also serve as growth promoters to accelerate the time to reach maturity as well as increase the body mass of animals. For all these reasons, antibiotics have become ubiquitous in society and have played an outsized role in shaping modern life.

However, despite numerous benefits, the drawbacks of antibiotics being so widely prevalent are now becoming apparent as well. With increasing exposure to antibiotics and corresponding selective pressure, bacteria have evolved to become resistant to many antibiotics, and antibiotic-resistant bacteria are widespread. As a result, existing antibiotics are losing their effectiveness to treat bacterial infections, and the problem is further compounded by the dearth of new antibiotics that have been discovered in recent years. In part, the problem is economic because pharmaceutical companies have had weak interest in developing new antibiotics due to low price points, however, the more pressing scientific issue is that the chemical space available for identifying and refining antibiotics is limited. There is growing recognition that society faces an impending post-antibiotic era [8], and hence, there is an urgent need to develop new classes of antibacterial agents that work against novel molecular targets.

To address this problem, antimicrobial lipids—single-chain lipid amphiphiles that destabilize bacterial cell membranes—are attractive candidates to become next-generation antibacterial agents for treating bacterial infections. Curiously, the antibacterial properties of antimicrobial lipids have been known since early reports by Dr. Robert Koch and colleagues in the late 1880s, when it was shown that fatty acids, a prominent class of antimicrobial lipid, inhibited growth of the *Bacillus anthracis* pathogen that causes anthrax [9]. A few decades later, Burtenshaw and colleagues showed that antimicrobial lipids are an important component of human skin’s innate immune system [10,11], lending credence to the possibility that exogenous addition of antimicrobial lipids would be medically opportune. Despite strong promise and demonstrated results, the prospects for antimicrobial lipids faded away by the late 1940s due to the emergence of antibiotics, but have received renewed attention amidst the growing impact of antibiotic-resistant bacteria. Indeed, one attractive feature of antimicrobial lipids is that it is difficult for bacteria to mutate to become resistant to them. As such, bacterial cell cultures can be grown in the presence of antimicrobial lipids (at sub-lethal concentrations) for at least one year, without signs of drug-resistant strains emerging [12].

Particular attention is drawn to two classes of antimicrobial lipid, namely fatty acids (hydrocarbon chains with a carboxylic acid functional group [13]) and monoglycerides (esterified adducts of a fatty acid and glycerol molecule). The motivation for studying these two classes of antimicrobial lipid arose from pioneering studies by Kabara and colleagues in the 1970s, which systematically investigated the antibacterial potency of medium-chain saturated fatty acids and monoglycerides with different chain lengths [14,15,16,17]. It was discovered that lauric acid (LA), which possesses a 12 carbon-long chain, had the most potent activity to inhibit growth of Gram-positive bacteria, and its monoglyceride derivative, glycerol monolaurate (GML), exhibited even stronger inhibitory activity than LA. Importantly, both LA and GML are Generally Recognized As Safe (GRAS) by the United States Food and Drug Administration [18] and abundant in nature. These factors have led to wide exploration of LA and GML for anti-infective applications [19,20,21], including application topics such as agriculture [22] and cosmetics [13,23,24]. Other antimicrobial lipids such as capric acid, which possesses a 10 carbon-long chain, and its monoglyceride derivative, monocaprin, have also received attention. While numerous studies have been conducted to empirically investigate the inhibitory properties of antimicrobial lipids, clarifying how the physicochemical properties of antimicrobial lipids influence biological activities remains an outstanding goal in many respects.

To date, the primary means of assessing the activity profile of an antimicrobial lipid has been to evaluate how treatment affects bacterial cell growth, with the minimum inhibitory concentration (MIC) of a test compound being defined as the drug concentration at which no visible growth of bacteria occurs. While such information provides insight into the scope and potency of an antimicrobial lipid, it does not reveal mechanistic information and there is growing interest to understand how antimicrobial lipids destabilize bacterial cell membranes. Biological assays have identified that antimicrobial lipids act as bacteriostatic (growth-inhibiting) or bactericidal (killing) agents depending on the drug concentration, target bacterium and other environmental factors [13,25]. To directly observe morphological effects on bacterial cell membranes, electron microscopy techniques have been utilized to image bacterial specimens after treatment with antimicrobial lipids [26,27,28,29,30,31,32]. While this approach provides visualization of membrane damage, very high concentrations of antimicrobial lipid are typically used (5–10 mM) and the bacterial cells can only be examined after treatment and sample fixation. Similar issues exist with atomic force microscopy experiments for examining the morphology of treated bacterial cells. In general, it is difficult to resolve molecular-level interaction kinetics when working with complex biological samples such as whole bacterial cells, thereby motivating the development of model systems.

To obtain insights into real-time interaction kinetics with a more focused approach, a variety of solution-phase model membrane platforms based on small unilamellar vesicles (SUVs) and large unilamellar vesicles (LUVs) have been employed in combination with measurement techniques such as dynamic light scattering in order to detect how antimicrobial lipids cause membrane destabilization via partial solubilization as well as membrane fission [33,34,35,36,37,38,39,40]. These model systems allow detailed characterization of membrane morphological changes by using well-controlled, simplified phospholipid compositions that mimic more complex biological membranes. Time-lapsed optical microscopy imaging of giant unilamellar vesicles (GUVs) has also enabled direct visualization of membrane morphological responses, including swelling, fusion, and fission behaviors [41,42]. As another option, supported lipid bilayers (SLBs) are two-dimensional phospholipid bilayers that have emerged as a particularly useful model membrane platform because they can be studied with a wide range of surface-sensitive measurement techniques, revealing insights into the mass, viscoelastic, fluidic, and morphological properties of SLB platforms. Indeed, one particular advantage of SLB platforms is that the two-dimensional phospholipid bilayer can remodel in response to an applied membrane strain, giving rise to three-dimensional morphological responses. Until recently, there was only scant attention to employ SLB platforms for investigating antimicrobial lipids, with only one study reporting the interactions between a long-chain fatty acid and single-component, zwitterionic phospholipid SLB platform and another study investigating the interaction between a short-chain monoglyceride and SLBs derived from bacterial cell membrane extracts [43,44,45]. Extending such approaches to investigate medium-chain saturated fatty acids and monoglycerides—representing the subset of antimicrobial lipids with the highest activity—is warranted in order to develop model systems for profiling the scope and potency of these antimicrobial lipids and a subject of active investigation. Moving beyond particular experimental approaches, there is active progress towards establishing comprehensive frameworks for correlating the physicochemical properties of antimicrobial lipids with their corresponding biophysical and biological activities.

Recognizing these possibilities, the goal of this review is to introduce the latest experimental approaches for characterizing how antimicrobial lipids destabilize phospholipid membranes within the broader scope of introducing current knowledge about the biological activities of antimicrobial lipids, testing strategies, and applications for treating bacterial infections. The contents of the review are organized into three major sections. First, a general background on antimicrobial lipids is provided that describes the structural classification, spectrum of antibacterial activity, and currently understood antibacterial mechanisms linked to membrane destabilization. Then, different experimental approaches to characterize antimicrobial lipids, including cell-based biological and model membrane-based biophysical measurement techniques, are presented. The major findings obtained by each experimental approach are critically summarized, and supplemented by discussion about how biological and biophysical approaches can be integrated to better understand how the physicochemical properties of antimicrobial lipids influence molecular self-assembly and concentration-dependent interactions with model phospholipid and bacterial cell membranes. Finally, it is described how antimicrobial lipids in free form and nanostructured assemblies can be employed for therapeutic applications, including when administered via systemic and topical administration routes. Taken together, the insights presented in this review underscore the utility of employing orthogonal measurement strategies to characterize the activity profile of antimicrobial lipids.

## 2. Antimicrobial Lipids

### 2.1. Classifications

Antimicrobial lipids are defined as single-chain lipid amphiphiles that interact with bacterial cell membranes and exhibit antibacterial activity. Fatty acids are a widely studied type of antimicrobial lipid, and are composed of a single saturated or unsaturated hydrocarbon chain and a carboxylic acid group on one end. As such, fatty acids are amphipathic molecules, with the hydrocarbon chain constituting the hydrophobic part while the carboxylic acid group is hydrophilic (either polar or anionic in aqueous solutions, depending on pH conditions). For example, the carboxylic acid groups of medium-chain fatty such as capric acid and LA, have pK_a_ values around pH 5 [46], and therefore the fatty acids are anionic (deprotonated carboxylic acid groups) around the physiological (blood) pH condition of 7.4. In addition to fatty acids, other derivatives have been reported to have antibacterial activity, with one prominent class being monoglycerides that are composed of a fatty acid connected with a glycerol molecule via an ester bond. Other synthetic versions are also possible such as related compounds with ether bonds, rendering such molecules impervious to bacterial lipases. Compared to fatty acids, monoglycerides bear the distinction of not having ionizable functional groups across relevant pH conditions, and hence, are nonionic molecules with neutral electrical charge properties and some degree of polarity.

**Figure 1:**
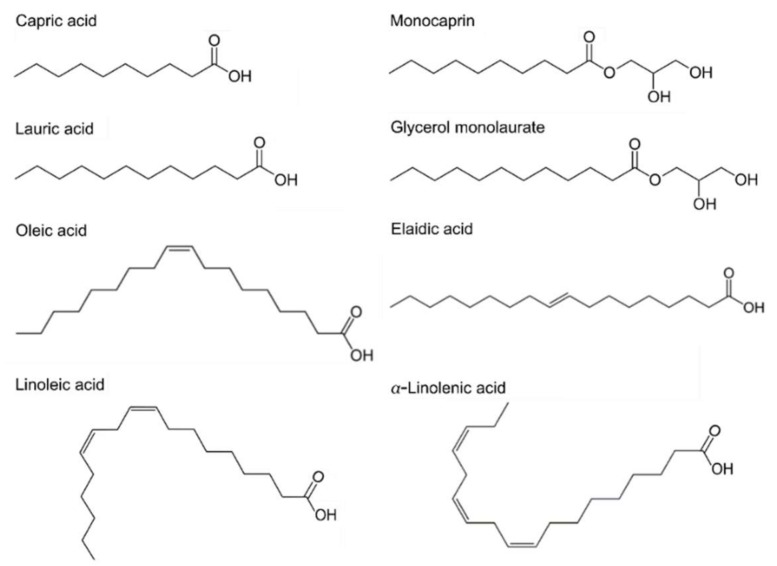
Chemical structures of fatty acids and monoglycerides. Saturated fatty acids; Capric acid (C10:0), LA (C12:0). Monoglycerides; Monocaprin (MG C10:0), Glycerol monolaurate (MG C12:0). Unsaturated fatty acids; Oleic acid (C18:1), Elaidic acid (*trans*-C18:1). Polyunsaturated fatty acids; Linoleic acid (C18:2), Linolenic acid (C18:3). C*x*:*y* is defined such that *x* is the number of carbons in the primary alkyl chain and *y* is the number of degrees of unsaturation.

Antimicrobial lipids are classified based on their chain lengths and degrees of unsaturation. Representative compounds with different molecular structures are presented in Figure 1. In biological systems, fatty acids typically possess an even number of carbon atoms between 4 and 28, although other odd-numbered versions are possible in select systems or synthetically produced. Fatty acids that are less than 8 carbon atoms long are defined as short-chain, while those with greater than 12 carbon atoms are long-chain fatty acids and medium-chain fatty acids have between 8 and 12 carbon atoms [13,47,48]. Another important parameter is the number of degrees of unsaturation. In saturated fatty acids, all the carbon atoms are linked by single covalent bonds, while unsaturated fatty acids have one or more double bonds (degrees of unsaturation) in the carbon backbone. Specifically, unsaturated fatty acids having more than one double bond are identified as polyunsaturated fatty acids. The presence of double bonds, including the number of them and their orientation (*cis*- or *trans*-) can lead to significantly different physicochemical properties of fatty acids, even among compounds having the same hydrocarbon chain length. Thus, classifying fatty acids and their derivatives is an important part of investigating trends in antibacterial activity with respect to molecular structure and shape. To this end, extensive microbiological studies have been conducted and unsaturated fatty acids with medium and longer chains typically show greater efficacy against Gram-positive bacteria than Gram-negative bacteria [49,50]. Some studies have also been reported that focus on assessing antibacterial potency in the presence of compounds with double bonds and related properties [27,50,51,52,53].

In past works, the potency of saturated fatty acids has also been examined as a function of hydrocarbon chain length [14,30,50,53,54,55]. By systematically investigating saturated fatty acids with hydrocarbon chains ranging between 6 and 18 carbons long, it was identified that LA, which has a 12-carbon long chain, exhibits the most potent activity to inhibit growth of Gram-positive bacteria, including *Staphylococcus aureus*, a major causative agent of bacterial skin infections as well as systemic ones. Its 1-monoglyceride derivative, GML, showed even greater potency against *S. aureus*, as indicated by a lower value of the minimum inhibitory concentration (MIC) in comparison to LA [14]. In addition, capric acid and its monoglyceride derivative, monocaprin, have saturated hydrocarbon chains that are 10 carbons long, and also have high antibacterial activity, especially against Gram-negative bacteria that are commonly associated with foodborne infections such as *Campylobacter jejuni* [56]. The connection between antimicrobial lipid and antibacterial spectrum and potency is further discussed in the next section.

### 2.2. Spectrum of Antibacterial Activity

Broad-spectrum inhibitory activity of antimicrobial lipids against algae, bacteria, fungi, protozoa, and virus has been reported for several decades [13,14,15,23,57,58,59]. The antibacterial potency of fatty acids and monoglyceride derivatives has been investigated extensively against a wide range of bacteria, including pathogenic strains such as methicillin-resistant *Staphylococcus aureus* (MRSA), as presented in Table 1. Extensive screening of the antibacterial activities of fatty acids was conducted by Kabara and colleagues in the 1970s, and helped to establish the modern-day field of antimicrobial lipids from a chemical perspective. Fatty acids with hydrocarbon chains ranging from 6 to 18 carbons long and selected derivatives with different functionalized headgroups were evaluated, resulting in the comprehensive identification of LA (C12:0) as the most potent antimicrobial lipid to inhibit growth of Gram-positive bacteria, as mentioned above. As a general principle, the esterification of a fatty acid to its corresponding monoglyceride derivative increases antibacterial activity [14,15,17]. Another supportive study for the high antibacterial potency of LA was conducted with different types of Gram-positive bacteria, and further demonstrated that unsaturated fatty acids with 18 carbon long chains—oleic acid (C18:1), linoleic (C18:2), and linolenic acid (C18:3)—have potent antibacterial activities as well [50].

Based on the findings from these pioneering studies, more selective and detailed studies focused on either specific bacterial type or particular antimicrobial lipids have been performed [27,52,60,61]. Specifically, interest on fatty acids and monoglycerides as potential therapeutic agents and/or preservatives against medically important pathogens led to the following studies of biomedical and food science relevance. Wang et al. investigated the efficacy of fatty acids and monoglycerides against *Listeria monocytogenes*, which is a Gram-positive bacterium that causes a series of food-borne infections. Of the tested fatty acids and monoglycerides, LA, linolenic acid, and GML had the strongest bactericidal activity at 10–20 µg/mL in brain heart infusion broth at pH 5. Interestingly, the bactericidal activity of the fatty acids depended on solution pH, showing increased activity at pH 5 as compared to pH 6, while the activity of the monoglyceride was not influenced by solution pH across this range [28]. This is consistent with the ionizable headgroup of fatty acids while monoglycerides are nonionic molecules.

Another target bacterium that is susceptible to fatty acids is *Helicobacter pylori*, which is a Gram-negative bacterium that can be pathogenic in the stomach or duodenum leading to diseases such as chronic gastritis, gastric ulcers, and stomach cancer. Petschow et al. tested a wide range of saturated fatty acids and corresponding monoglycerides with chain lengths from 4 to 17 carbons against *H. pylori* and observed the following trend in bactericidal activities. Monoglycerides with 10–14 carbon long chains showed appreciable bactericidal efficacy at 1 mM concentration and had a lower tendency of spontaneous resistance development, while LA was the only saturated fatty acid that had bactericidal potency under the tested conditions [62]. Similarly, the anti-H. pylori activity of LA and GML was demonstrated by Sun et al., in which case selected unsaturated fatty acids were evaluated along with a range of saturated fatty acids (4–16 carbon chains) and monoglycerides (12–16 carbon chains) [54]. LA completely killed *H. pylori* at minimum bactericidal concentration (MBC) of 1 mM, and GML showed even more potent activity than LA with a lower MBC value around 0.5 mM. Additionally, it was identified that, among unsaturated fatty acids, myristoleic (C14:1) and linolenic acid have the most potent activity against this bacterium [54]. Bergsson and colleagues also assessed inhibitory activity against Gram-negative bacteria, including *H. pylori*, for a wide range of fatty acids and corresponding monoglycerides [64]. Interestingly, the tested compounds that had hydrocarbon chains between 10–16 carbons long exhibited inactivation of *H. pylori* at 10 mM concentration, however, there was no significant activity observed against *Salmonella* species and *Escherichia coli*. Bergsson et al. conducted additional studies in order to characterize the antibacterial potency of selected antimicrobial lipids against different bacteria, and it was demonstrated that monocaprin (MG C10:0) is the most potent bactericidal agent against *Chlamydia trachomatis* and *Neisseria gonorrhoeae* [29,63]. Additional studies have been reported investigating the effects of fatty acids (with chains of 2 to 18 carbon length) on Gram-negative bacteria, including *E. coli* [65] and *Salmonella* species [66], and demonstrated that caprylic acid (C8:0) has particularly high antibacterial activity against these bacteria. Antibacterial activity of caprylic acid and its monoglyceride, monocaprylate, were also identified against fish pathogens including *Edwardsiella* species by Kollanoor and colleagues [69]. Of particular note, caprylic acid and monocaprylate have showed potent inhibitory activity against important foodborne pathogens, including *E. coli* O157:H7 [73,74,75,76,77], along with *Salmonella* species [78,79], *L. monocytogenes* [80,81], and mastitis pathogens [82].

Of relevance to skin infection, *S. aureus* and MRSA are a leading cause of skin and soft-tissue infections, including acute bacterial skin and skin structure infections. Within this scope, Kitahara et al. investigated a range of saturated fatty acids against conventional *S. aureus*, methicillin-susceptible *Staphylococcus aureus* (MSSA) and MRSA, and it was identified that LA is the most potent saturated fatty acid against MSSA and MRSA [19]. Potent inhibitory activity of GML has also been reported against *S. aureus* in the following studies. Lin et al. demonstrated that GML killed the bacteria effectively both in vitro and in vivo and reduced toxic shock syndrome [83]. Another in vivo study performed by Preuss et al. confirmed the antibacterial activity of GML against *S. aureus* infection [84]. In addition, Nakatsuji et al. explored the feasibility of employing LA to treat a mouse model infection caused by another Gram-positive bacterium, specifically *Propionibacterium acnes* [20]. Hence, multiple antimicrobial lipids have shown in vivo therapeutic potential, and there is also growing interest to investigate combinations of several antimicrobial lipids that have antibacterial potency against specific bacteria in order to achieve synergistic and broad-spectrum effects [85,86,87,88,89].

### 2.3. Mechanisms of Antibacterial Activity

The mechanism(s) of antibacterial activity of fatty acids and monoglycerides have been explored using diverse experimental methods to identify that they mainly target bacterial cell membranes and interrupt crucial processes involved in cellular protection and function. The membrane-lytic behavior of fatty acids and monoglycerides stem from their amphipathic properties, leading to overlapping sets of biophysical phenomena including membrane destabilization and pore formation. In particular, membrane-destabilizing activity causes increased cell permeability and cell lysis, leading to inhibition of bacterial cell growth (bacteriostatic action) or cell death (bactericidal action).

**Figure 2:**
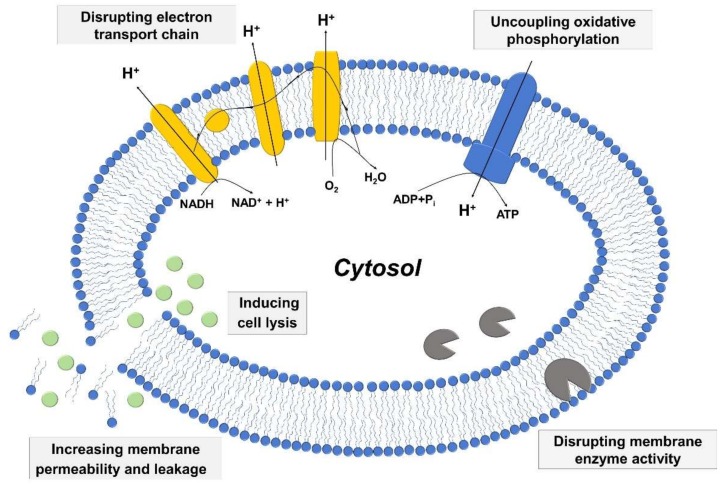
Schematic representation of mechanisms behind the antibacterial activity of fatty acids and monoglycerides.

Among vital processes involving bacterial cell membranes, two of the most important ones involve the electron transport chain and oxidative phosphorylation, which are essential for energy production in bacterial cells. The two processes are interconnected, and fatty acids have the potential to disrupt the electron transport chain process by binding to electron carriers or altering membrane integrity as well as interfering with oxidative phosphorylation by decreasing the membrane potential and proton gradient. Moreover, fatty acids can directly inhibit membrane enzymes such as glucosyltransferase, presumably due to similar molecular structures of fatty acids with known small molecule inhibitors [90,91], and also target other membrane-associated proteins as well [92]. Figure 2 presents a schematic illustration describing key antibacterial activities of fatty acids and monoglycerides that relate to targeting bacterial cell membranes. The details of the mechanistic processes can be classified based on the relationship between the following three aspects: (i) increased membrane permeability and cell lysis, (ii) disruption of electron transport chain and uncoupling oxidative phosphorylation, and (iii) inhibition of membrane enzymatic activities and nutrient uptake.

#### 2.3.1. Increased Membrane Permeability and Cell Lysis

The interaction of antimicrobial lipids with bacterial cell membranes can destabilize the membrane and increase membrane permeability, thereby inducing leakage of cytosolic contents. In extreme cases, the increased permeability and corresponding membrane destabilization can eventually lead to cell lysis, as documented in numerous experimental studies. For example, Chamberlain et al. observed that when *S. aureus* cell membranes were treated with oleic acid, there was an increase in membrane permeability as determined by polarized fluorimetry. Oleic acid treatment lowered the polarization value, which indicated increased membrane fluidity and led to cell death [93]. Similarly, Greenway et al. reported that linolenic acid treatment caused the damage of *S. aureus* cell membranes [61]. In that case, membrane leakage after linolenic acid treatment was detected by measuring the release of biomolecules (e.g., glutamic acid) from bacteria, and absorbance spectroscopy experiments at 260 nm wavelength were conducted to measure the release kinetics, which is correlated with the extent of bacterial cell membrane leakage [94]. As such, the measurements showed that linoleic acid inhibits the growth of *S. aureus* by inducing a marked increase in membrane permeability—interpreted as pore formation—and this increased permeability further inhibits macromolecular synthesis and coupling of the electron transfer chain [61]. Boyaval et al. also demonstrated that the strong membrane-disruptive activity of linoleic acid causes leakage against *Propionibacterium freudenreichii* subsp *shermanii* [95]. In particular, linoleic acid interrupted bacterial cell growth by increasing membrane permeability, as measured by monitoring potassium efflux and transmembrane electrical potential. Increased permeability resulted in an increase in K^+^ efflux and a decrease in membrane potential. Beyond biochemical readouts, many studies have reported using electron microscopy to investigate the membrane-disruptive activity of fatty acids against various bacterial cell types [26,27,28,29,31,32]. In most of these studies, high concentrations of antimicrobial lipid in the millimolar range were used and hence, the membrane morphological damage was quite extensive and observed post-treatment. In addition, the membrane-disruptive activities of monoglycerides against different bacterial cell types have been reported [28,29]. Regarding direct observation of cell lysis, Carson et al. reported that certain unsaturated fatty acids, namely oleic acid or linoleic acid, can cause lysis of *Streptococcus faecalis* [96]. Thompson et al. further showed how treatment of *H. pylori* with linolenic acid induces cell lysis, as revealed by electron microscopy [97].

#### 2.3.2. Disrupting Electron Transport Chain and Uncoupling Oxidative Phosphorylation

The electron transport chain is a key complex that consists of electron carriers and produces the energy source, adenosine triphosphate (ATP), and is coupled to oxidative phosphorylation through the ATP synthase, an enzyme that synthesizes ATP. The electron transport chain and ATP synthase are both located at the inner membrane of Gram-positive and Gram-negative bacteria [98]. Electrons are transported from one carrier to another until reaching the final electron accepter, which is oxygen [99]. The electron transport process is accompanied by proton (H^+^) transfer from the cytosol to outside of the cell, creating a proton gradient across the cytoplasmic membrane and increasing the membrane potential that provides an energy source to produce ATP via ATP synthase. When one of the steps in the electron transport chain and oxidative phosphorylation is interrupted, it is difficult for bacterial cells to have sufficient energy to function, which leads to inhibition of cell growth and eventually cell death. By measuring oxygen uptake with a Clark type oxygen electrode setup, interruption of the electron transport chain can be assessed. Following this approach, Galbraith et al. demonstrated that LA and myristic acid were the most effective saturated fatty acids to inhibit oxygen intake for *Bacillus megaterium* and *Pseudomonas phaseolicola*, while linoleic acid was the most effective unsaturated fatty acid and was active at much lower concentrations (greater potency) than saturated fatty acids [100]. In another related study, Greenway et al. investigated the disruption of the electron transport chain in *S. aureus* in response to linoleic acid treatment [61]. Moreover, Sheu et al. investigated the effect of fatty acid treatment on *Bacillus subtilis* by measuring oxygen uptake and ATP concentrations, and demonstrated that the interaction of fatty acids with the bacterial cell membrane decreased membrane integrity, resulting in decreases in oxygen uptake and ATP levels [101]. While it is difficult to directly measure the effect of fatty acid treatment on electron carrier transport in live bacteria, it is noteworthy that Peters et al. investigated electron transport in intact chloroplasts, demonstrating that palmitoleic acid (C16:1 fatty acid) mainly inhibited photosystem (PS) II by up to 90% as part of the electron transport system [102]. Although the latter study was not conduced on bacterial cell membranes, the results still provide useful insight to understand how membrane destabilization caused by antimicrobial lipids can cause severe detriments to electron transport carriers and downstream biochemical processes.

#### 2.3.3. Inhibiting Activity of Bacterial Enzymes

Another significant mechanism by which antimicrobial lipids affect bacterial cell membranes is inhibiting the activity of membrane-associated enzymes, and there have been numerous studies exploring the effects of fatty acid treatment on specific bacterial cell enzymes. For example, Kurihara et al. observed that certain fatty acids inhibit glucan production catalyzed by glucosyltransferase (GTase) from *Streptococcus sobrinus*, which leads to inhibition of bacterial cell growth. GTase is an important transmembrane protein that mediates glucan production in bacterial cells and is mainly produced by *Streptococcus mutans* and *Strep. sobrinus*. Significantly, unsaturated fatty acids such as oleic acid, linoleic acid, and arachidonic acid (C20:4) exhibit even stronger inhibition of GTase activity, while there is an almost negligible effect caused by saturated fatty acids [90]. This finding is significant because it supports that antimicrobial lipids with different physicochemical properties can have unique modes of interacting with bacterial cell membranes. Another study also showed GTase inhibition by oleic acid, supporting that the antibacterial activity of unsaturated fatty acids is related, at least in part, to inhibiting membrane-associated enzymes [91].

Additionally, it has been reported that fatty acids and related derivatives can inhibit bacterial growth by inhibiting fatty acid biosynthesis. Indeed, fatty acids play important roles in bacteria because they are precursors of important cellular materials. Zheng et al. demonstrated that unsaturated fatty acids, including linoleic acid, show antibacterial activity against *S. aureus* by inhibiting bacterial enoyl-acyl carrier protein reductase (Fabl), which is an important enzyme involved in the fatty acid elongation process [103]. Similarly, the antibacterial activity of medium-chain saturated and unsaturated fatty acids against *S. aureus* was investigated in terms of inhibiting fatty acid synthesis, and the results indicated that, among the tested compounds, α-linolenic acid was particularly inhibitory [104]. To what extent the effects of antimicrobial lipids on bacterial enzymes are direct or indirect remain to be understood in the broader context of membrane destabilization processes, while it is clear that the effects of antimicrobial lipids on bacterial membranes can inhibit key enzymatic activities. Within this scope, it is particularly intriguing that unsaturated fatty acids affect bacterial enzymes, while saturated fatty acids typically have negligible effect on the same enzymes. Such findings motivate the overall motivation to establish measurement platforms for characterizing the mechanism of action and potency of antimicrobial lipids acting against phospholipid membranes, and to draw correlations with biological activities. In the following section, the main experimental techniques to characterize antimicrobial lipids are presented, including biological and biophysical methods.

## 3. Experimental Approaches to Characterize Antimicrobial Lipids

The inhibitory activity of antimicrobial lipids has been widely investigated by employing biological approaches based on anti-infective evaluation of bacterial specimens. Antibacterial susceptibility tests such as MIC and minimum bactericidal concentration (MBC) assays are commonly used. The MIC assay is a method of determining the minimum concentration of a test compound that inhibits bacterial growth, thereby enabling rapid screening of the antibacterial susceptibility of antimicrobial lipids against a target bacterium. A more detailed understanding about the mode of action of a test compound can be obtained by determining the MBC value, which is defined as the minimum concentration of a test compound to completely kill a target bacterium. Although both MIC and MBC assays facilitate empirical evaluation of the antibacterial potency of a drug candidate, and inform about the antibacterial spectrum of a antimicrobial lipid, the assays do not probe how antibacterial lipids destabilize bacterial cell membranes. To address such questions, electron microscopy has been widely used to visualize the antibacterial activity of antimicrobial lipids by observing morphological changes of bacterial specimens after treatment with antimicrobial lipids. While useful to look at gross morphological changes, this approach has limitations because it typically requires high lipid concentrations and the bacterial cells are analyzed after treatment and sample fixation. To facilitate real-time monitoring of the membrane interactions involving antimicrobial lipids, one of the most useful approaches has focused on developing model membrane platforms to investigate molecular-level interactions. The different experimental techniques used in biological and biophysical studies are summarized in Table 2. Following this line, details about biological and biophysical approaches are introduced in this section.

### 3.1. Anti-Infective Evaluation of Bacterial Specimens

#### 3.1.1. Growth Inhibition Assays

One of the most widely used experimental assays to determine the antibacterial activity of test compounds involves determining the MIC of a compound that is able to inhibit bacterial growth. Formally, the MIC is defined as the lowest concentration of test compound that inhibits observable bacterial growth, and MIC values are often used as quantitative indicators of the relative potency of new antibacterial agents [105]. The agar and broth dilution methods are the most common protocols for determining MIC values [106,107].

Kabara and colleagues conducted pioneering studies to evaluate the MIC values of fatty acids and monoglycerides against a wide range of bacteria by using the broth dilution method, and the recorded MIC values are summarized in Table 3 and Table 4 for fatty acids and monoglycerides, respectively. In addition, Galbraith and Nakatsuji also tested fatty acids against other bacteria of interest. Of note, Kitahara et al. reported an unconventional method to determine MIC values based on measuring oxygen levels using an oxygen electrode sensor named DOX-96 [19]. As mentioned above, saturated fatty acids and monoglycerides with 12-carbon long chains exhibited the most potent activity to inhibit the growth of Gram-positive bacteria, with particularly high antibacterial activity against the methicillin-resistant *Staphylococcus aureus* (MRSA) strain which causes serious acute skin infections in humans [108]. In general, monoglycerides have lower MIC values than fatty acid equivalents against different bacteria.

While the trend in MIC results are generally reproducible in terms of evaluating releative potency, reported MIC values are sometimes quite different for the same test compound against the same bacteria, due to variations in experimental conditions. On one hand, multiple reports by Kabara and colleagues showed similar MIC values for the same compound (prepared in roughly equivalent ways in the two studies) against *S. aureus*, as determined by the broth dilution method [14,15]. On the other hand, the MIC values obtained for the same compound can also often be quite variable depending on the solution condition. For example, using the same measurement method, it has been reported that the MIC value of LA against *S. aureus* varied around 500–1000 µM and GML around 31–125 µM depending on the preparation method in PBS solution or Mueller-Hinton (MH) Broth [109]. Even greater variations are reported in the literature when considering different experimental methods. Additionally, antimicrobial lipids are particularly sensitive to temperature [110,111] and pH [21,28,31,54,60,65,66,112,113] as well, and the experimental conditions should be designed and modified appropriately depending on the type of test compound.

When it comes to antimicrobial lipids, another key issue is compound solubilization and how it affects the molecular self-assembly of compounds and resulting potency observed in the MIC experiments. Comparing the MIC values obtained for LA (C12:0) against *S. aureus* in studies by different groups reveals that the potency of a single compound against a single bacterium can vary by over 500-fold. In the two studies, it was noticed that different organic solvents were used for solubilizing LA molecules, and the highest test concentrations contained 1% ethanol or 5% dimethyl sulfoxide (DMSO) in the two studies, respectively. As such, the presence of organic solvents or other environmental factors likely influences the concentration-dependent molecular self-assembly of antimicrobial lipids in bulk solution, and such variations are only reflected in the MIC readout on the basis of how the compounds inhibit bacterial growth. For this reason, MIC assays provide an initial empirical assessment of antibacterial activity while additional methods are needed to further characterize the mechanism of action of a compound.

#### 3.1.2. Infectivity Assays

While MIC readouts assess the capacity of a drug candidate to inhibit bacterial growth, the information obtained does not provide direct information about whether or not treatment with a drug can directly inactivate a bacterium by way of killing, e.g., lytic effect. To address such questions, which are particularly relevant to consider for antimicrobial lipids since they are membrane-lytic agents, other methods have been devised and involve determining the MBC, which is defined as the lowest concentration of antimicrobial agent to kill a target bacterium. Formally, the MBC value is the lowest concentration of a test compound at which ≥99.9% of the initial bacterial inoculum are killed within 24 h [114]. Normally, if the determined MBC value is no more than 4 times greater than the MIC value of a test agent, then the agent is considered to have bactericidal activity. Otherwise, the candidate is considered to have principally bacteriostatic activity.

There are many studies investigating the bactericidal activity of fatty acids and monoglycerides by determining MBC values. Sun et al. reported the MBC values of the C12:0 saturated fatty acid and monoglyceride pair, LA and GML, against *H. pylori*. The MBC values of LA and GML were 1 mM and 0.5 mM, respectively [54]. Wang et al. tested LA, linolenic acid, and GML and showed that all three compounds exhibit bactericidal effects against *L. monocytogenes*. LA, linolenic acid, and GML had MBC values of 10, 20, and 10 µg/mL, respectively, at the pH 5 condition. Interestingly, at higher pH conditions around 6, the MBC values of LA and linolenic acid increased to 20 and 100 µg/mL, respectively, while the MBC value of GML remained unchanged at 10 µg/mL and was not influenced by the change in solution pH [28]. In addition, the MBC value of LA against *P. acnes* was also determined to be 60 µg/mL [20]. Direct comparison of MBC values for LA and GML against *S. aureus* was also carried out, and it was reported that the MBC values for LA and GML are 50 mM and 0.25 mM, respectively [21]. This finding indicated that GML has about a 200-fold lower MBC value and appreciably greater bactericidal activity against *S. aureus*. Without directly mentioning the MBC concept, Petschow et al. counted the number of viable bacterial cells by colony-forming unit (CFU) enumeration after treatment with fatty acids or monoglycerides, and determined that LA is the only tested saturated fatty acid that showed bactericidal activity against *H. pylori*. In particular, treatment of *H. pylori* with 1 mM LA for one hour yielded a greater than 4 log_10_ CFU/mL reduction. Among tested monoglycerides, monocaprin and GML also showed a bactericidal effect in similar fashion [62].

Although MBC is an excellent metric to evaluate antibacterial activity and to help understand mechanistically whether or not a test compound completely kills or inhibits the growth of a bacterium, MBC values alone can also be variable depending on the technical format and experimental conditions, in analogous fashion to the challenges facing MIC determinations. Ultimately, both MIC and MBC values provide insight into the scope and potency by which antimicrobial lipids affect the infectivity of bacterial species, and can guide structure-activity relationships at the biological level. However, such assays do not provide information about how antimicrobial lipids destabilize bacterial cell membranes and hence, there is limited room to explore optimization strategies or understand the physicochemical basis underpinning the scope and potency of particular compounds. Hence, there has been more direct experimental methods to observe the interactions between antimicrobial lipids and bacterial cells.

#### 3.1.3. Electron Microscopy

**Figure 3:**
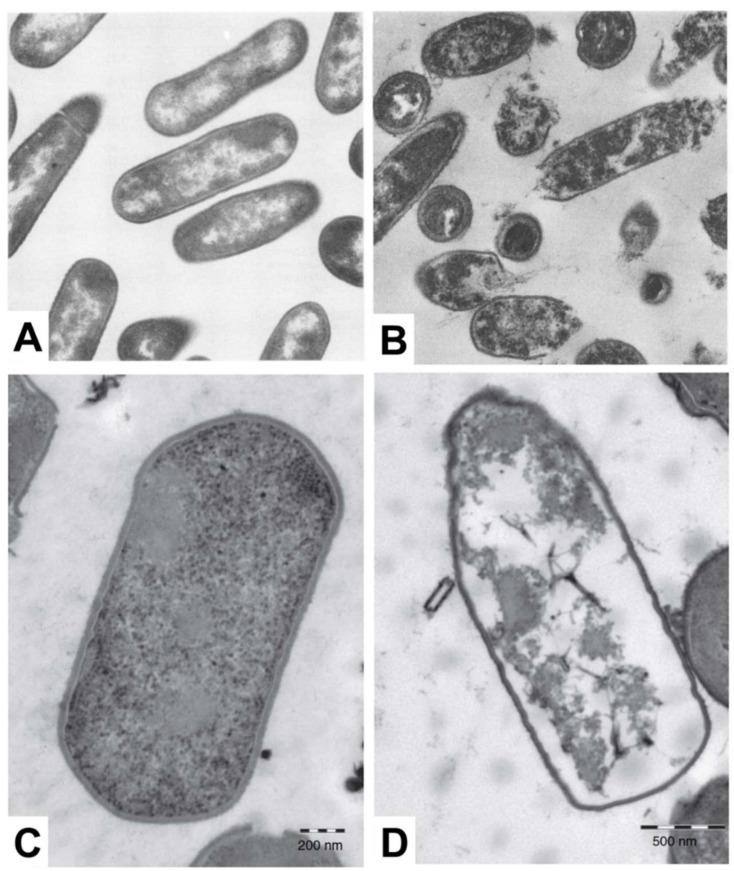
TEM micrographs show the effect of treating bacterial cells with fatty acids and monoglycerides. *L. monocytogenes* cells that are (**A**) untreated or (**B**) treated with 50 µg/mL GML (magnification ×44,080) and *C. perfringens* cells that are (**C**) untreated or (**D**) treated with 1 mg/mL LA. Reproduced with permission from [28,31].

Electron microscopy is a popular measurement technique for investigating the morphological structure of bacterial cell samples and can be utilized to study the effects of antimicrobial lipid treatment [115]. Among the different techniques, scanning electron microscopy (SEM) is useful to characterize the cell surface moprhology, while transmission electron microscopy (TEM) facilitates characterization of surface morphology along with the density of inner cytoplasmic constituents [116,117]. When electron microscopy techniques were first introduced for studying antimicrobial lipids, most related studies utilized TEM for imaging the effects of treating bacteria with fatty acids. A summary of key observations made by electron microscopy analysis is reported in Table 5, including treatment conditions with antimicrobial lipid and corresponding target bacterium. In the late 1970s, Speert et al. explored the detailed mechanism of bactericidal activity underpinning how oleic acid affects Streptococcus group A. Based on TEM imaging measurements, it was observed that cytoplasmic contents become disorganized, including vacuolization in the cytosol and condensation of nucleoids [26]. Following this work, Knapp et al. observed significant membrane disruption of *N. gonorrhoeae* when the bacterium was treated with 10 µM arachidonic acid, as revealed in TEM experiments [27]. All of the cytoplasmic contents appeared to leak out from morphologically deformed *N. gonorrhoeae* cells. In contrast, however, similar treatment did not affect the surface morphology of *S. aureus* cells, while there was still disruption and condensation of cytoplasmic contents in the latter case. There are also some TEM studies that investigate how monoglycerides affect bacterial cells. Based on extensive screening of the antibacterial activity of fatty acids and monoglycerides, Wang et al. identified that linolenic acid and GML are the most potent fatty acids and monoglycerides, respectively, against *L. monocytogenes* and the antibacterial effects were further investigated by TEM. Interestingly, cell lysis occurred upon treatment with 50 µg/mL GML along with leakage of cytoplasmic contents, as presented in Figure 3A,B. On the other hand, upon treatment with 200 µg/mL linolenic acid, only irregular changes in surface morphology were detected without cell lysis [28]. The antibacterial activity of monocaprin against bacterial cells has also been studied by Bergsson and colleagues. Upon treatment with 10 mM monocaprin, the elementary body form of *C. trachomatis* became shrunken through morphological deformation [29]. Subsequent studies exploring the potency of monocaprin were carried out on Streptococcus group B, first by using SEM followed by TEM experiments. Significant changes in surface morphology, including size and shape, were not observed by SEM, while TEM further revealed that the bacterial cell membrane and granule structures disappeared after monocaprin treatment, although the cell wall remained intact [30]. Similarly, LA also disrupted and separated cell membranes in *Clostridium perfringens* leading to cytoplasmic disorganization without causing significant changes in cell wall structure, as presented in Figure 3C,D [31]. Using SEM, Shin et al. showed that after treating *S. aureus* and *Pseudomonas aeruginosa* with eicosapentaenoic acid, severe morphological disruption was observed with rough surface features becoming apparent [32].

Taken together, a vast body of knowledge about how antimicrobial lipids affect bacterial cells has been elucidated by electron microscopy techniques. Among the findings, it is evident that there is a spectrum of ways in which antimicrobial lipids can perturb bacterial cells, including disruption of cell membranes and related effects such as loss of cytoplasmic contents. However, at the same time, distilling the empirical insights into general principles describing how antimicrobial lipids affect bacterial cell membranes remains difficult to achieve with electron microscopy results. Aside from the high lipid concentrations that are commonly tested, the low measurement throughput, and the divergent structural and compositional properties of different tested bacterial species, it is only possible to examine the effects of treatment after bacterial specimens have been fixed and therefore it is not possible to monitor changes in morphological properties in real-time. To address such needs, a wealth of complementary biophysical techniques has been developed based on employing model membrane systems, as discussed in the next section.

### 3.2. Biophysical Approaches with Model Membrane Platforms

#### 3.2.1. Solution-Phase Liposomes

Solution-phase liposome assays have been developed to monitor the interaction kinetics between antimicrobial lipids and phospholipid membranes, by measuring changes in the size distribution of liposomes in bulk solution. One advantage of this approach is that the phospholipid compositions of the liposomes are highly simplified and therefore offer excellent control to understand how specific factors influence resulting interaction processes. The biophysical approach therefore provides a complementary approach to look at how fundamental parameters such as compound concentration affect interaction processes, while it should be noted that the simplified lipid compositions do not fully mimic the more complex membrane structures surrounding bacterial cells. Nevertheless, model membranes provide a useful tool to obtain key insights into compound-specific membrane interaction processes and compound potency. Among the measurement options, dynamic light scattering is utilized as an ensemble-average measurement technique to determine the size and polydispersity of liposomes in bulk solution, including size changes after treatment with antimicrobial lipids. In addition, electron microscopy is employed to visualize how antimicrobial lipids induce morphological changes on individual liposomes.

To date, several studies have been reported that perform complementary electron microscopy and dynamic light scattering experiments and focused on oleic acid/oleate compositions, as presented in Table 6. By using the thin film hydration method, liposomes can be prepared across a range of sizes, which are classified as small unilamellar vesicle (SUVs) up to 100 nm diameter, large unilamellar vesicles (LUV) with diameters between 100 and 400 nm [118], and giant unilamellar vesicles (GUVs) with diameters above 1 µm [119]. The studies were typically related to a “matrix effect” describing the phenomenon that, in the presence of preexisting liposomes, the rate of forming new liposomes—as induced by the fatty acids—is appreciably accelerated, and the size distribution of the newly formed liposomes depended on the size of the preexisting liposomes and/or the molar ratio of fatty acids added to the liposomes [33,120]. The first direct observation of the matrix effect was made when Blöchliger et al. showed that autocatalysis of oleate in aqueous solution was accelerated to form new liposomes in the presence of preexisting oleic acid/oleate liposomes [120]. In addition to the presence of the preexisting liposomes, the size distribution of newly formed, mixed liposomes appeared within a narrow size range and the results showed a similar size distribution to the preexisting liposomes (e.g., 50 or 100 nm diameter), while the newly formed liposomes from oleate itself without the preexisting liposomes had a broad range of sizes ranging from 50 nm to 1.5 µm diameter.

**Figure 4:**
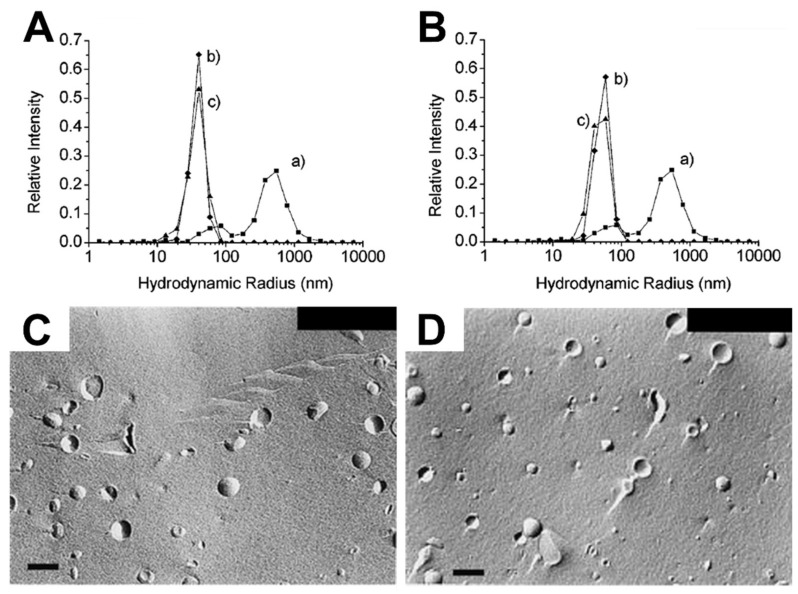
DLS measurements and complementary freeze-fracture electron micrographs measuring the interaction of fatty acids with SUVs and LUVs. DLS-measured size distribution curves of POPC lipid vesicle extruded with (**A**) 50 nm, (**B**) 100 nm diameter pore filters before (curve b) and after (curve c) oleate addition, and freeze-fracture micrographs of (**C**) preformed 180-nm diameter Egg PC vesicles and (**D**) Egg PC/oleate (1:1) vesicles. The scale bars are 200 nm. Reproduced with permission from [36] (Copyright 2003, American Chemical Society) and [38].

There are also numerous studies reporting the “matrix effect” when using phospholipid liposomes [33,35,36,37,38,39,40]. Lonchin et al. studied how different molar ratios of oleate added to preexisting POPC (1-palmitoyl-2-oleoyl-*sn*-glycero-3-phosphocholine) liposomes influence the size distribution of newly formed liposomes. Adding a large amount of oleate to POPC liposomes induced the formation of larger mixed POPC/oleate liposomes with increased polydispersity, while similar sizes of mixed liposomes occurred when a lower amount was added [33]. Berclaz et al. provided further evidence showing how the size distribution of newly formed liposomes varies depending on the amount of oleate added to preexisting phospholipid liposomes, as determined by cryogenic TEM (cryo-TEM) analysis [34,35]. Using DLS, Rasi et al. also investigated the formation of newly formed liposomes in response to the matrix effect caused by oleate treatment of POPC liposomes. The size distribution curves before (curve b) and after (curve c) oleate addition to preexisting 50 and 100 nm POPC SUVs were quite similar, as presented in Figure 4A,B [36]. Chungcharoenwattana and colleagues confirmed the matrix effect by employing a particular system, gel filtration chromatography combined with DLS, to measure the detailed size distribution of newly formed phospholipid/oleate liposomes across individual elution fractions [37]. Using freeze-fracture electron microscopy, it was also explored how adding different amounts of oleate to preexisting phospholipid liposomes affects the resulting liposome size distribution, whereby the addition of a small amount caused a narrower size distribution of newly formed mixed liposomes than the addition of a large amount, as shown in Figure 4C,D [38]. At a mechanistic level, it has been suggested that fatty acid interactions cause membrane fission and partial solubilization, giving rise to the matrix effect and related phenomena [38,39]. The effects of adding various fatty acids to preexisting DMPC (1,2-dimyristoyl-*sn*-glycero-3-phosphocholine) or POPC SUVs have also been determined by turbidity measurements based on UV/Vis spectrophotometry experiments [40]. The fatty acids exhibited rapid incorporation into the preexisting liposomes, followed by the formation of mixed phospholipids/fatty acid liposomes through size growth and subsequent fission. These changes were determined by noticing that the turbidity increased dramatically in the presence of preexisting liposomes as compared to when preexisting liposomes were absent. Hence, solution-phase liposomes provide a useful platform for investigating the types of morphological changes that occur when antimicrobial lipids are added to phospholipid membranes. Typically, the data analysis focuses on antimicrobial lipid:phospholipid ratio and the experimental readouts are largely based on gross morphological changes and changes in size distribution. Hence, the measurements provide indications that membrane interactions are occurring, however, tracking details of specific membrane interaction processes still require additonal biophysical tools.

#### 3.2.2. Giant Unilamellar Vesicle

To directly visualize membrane morphological changes induced by antimicrobial lipids and related compounds, giant unilamellar vesicles (GUVs) in solution-based systems have been utilized to monitor interaction kinetics. Typically, the GUVs are greater than 10 µm diameter and are hence, able to be studied by optical microscopy, including fluorescence-based labeling of phase-sensitive membrane components for visualizing phenomena such as phase separation [41,42,121,122,123,124,125,126,127]. Individual GUVs are directly studied and a wide amount of information about morphological behavior, including fluctuations and membrane fission/fusion, can be monitored in real-time. Pioneering interactions studies showed that after treating GUVs with representative membrane-active, nonionic surfactants, membrane morphological responses could be tracked in real-time. Tamba et al. observed membrane disruption and leakage in response to treatment with Triton X-100 and octylglucoside [125]. Mavčič et al. assessed the influence of another nonionic surfactant, octaethyleneglycol dodecylether (C_12_E_8_), on GUVs, and dynamic morphological responses such as the shape transformation from tubular to spherical formations depending on the C_12_E_8_ concentration [126].

**Figure 5:**
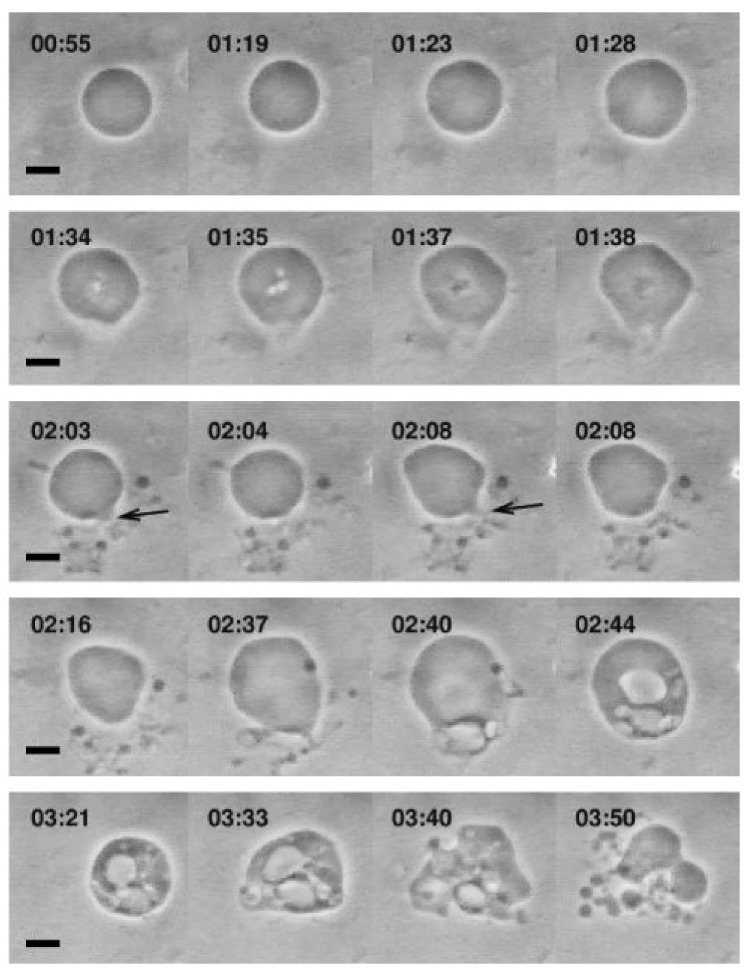
Optical micrographs showing the morphological responses of POPC GUVs that occur upon treatment with 0.8 mM oleic acid solution. The scale bars are 10 µm. Reproduced with permission from [41].

The interaction of various single-chain lipid amphiphiles with GUVs has also been investigated. Inaoka et al. investigated membrane fission in GUVs that occurred upon treatment of lysophosphatidylcholine (LPC) containing 16-carbon long alkyl chains at low concentration, e.g., 2 µM LPC, along with varying the membrane composition and amphiphile concentrations [127]. Tanaka et al. also observed similar membrane fission behavior with interesting shape changes occurring as well [125]. In that study, LPC molecules with different chain lengths ranging from 10 to 16 carbons long were tested against cholesterol-containing GUVs, and the resulting shape changes varied from prolate to asymmetrical spherical shape and membrane fission occurred above a corresponding threshold concentrations for each LPC (which decreased with increasing chain length). Furthermore, Peterlin et al. monitored morphological responses of 1-palmitoyl-2-oleoyl-*sn*-glycero-3-phosphocholine (POPC) GUVs upon treatment with 0.8 mM oleic acid solution, under which condition mixed oleic acid/oleate liposomes form [41]. Upon treatment, the GUVs started growing followed by various responses such as membrane invaginations, evaginations and budding, and finally creating small budding liposomes that were attached to the mother GUV, as presented in Figure 5. Recently, Mally et al. utilized phase-contrast microscopy to characterize the interactions between oleic acid and GUVs, revealing how fatty acid insertion causes an increase in GUV size followed by bursting [42]. A physical model was developed to explain the results, with particular focus on the role of membrane strain in triggering the burst after reaching a critical threshold. As such, at an observational level, GUV platforms have provided a useful tool to study the interactions between antimicrobial lipids and phospholipid membranes, and developing preliminary mechanistic models to explain the basis for membrane interactions, including concentration-dependence.

#### 3.2.3. Supported Lipid Bilayers

Expanding beyond solution-phase studies, the supported lipid bilayer (SLB) platform has emerged as a promising measurement platform to study the mechanism of action of antimicrobial lipids, including fatty acids and monoglycerides, interacting with phospholipid membranes. SLB platforms are composed of two-dimensional phospholipid bilayers that are supported on a hydrophilic support and the bilayer-substrate interaction stabilizes the model membrane [128] while preserving key functional features of membranes in general. One particular advantage of SLB platforms is that they can be studied by a wide range of surface-sensitive measurement techniques, thereby allowing detailed investigation of the interaction kinetics from multiple perspectives, including binding mass, change in viscoelastic properties, and membrane fluidity.

As mentioned above, the insertion of antimicrobial lipids and other single-chain lipid amphiphiles into phospholipid bilayers induces membrane strain, and the bilayers can undergo membrane remodeling in order to response to the applied strain. Staykova et al. observed how osmotic pressure changes can generate membrane strain in SLB platforms and presented a physical model to describe the resulting remodeling processes [129]. Specifically, the SLB platform responds by deforming to form spherical or tubular shapes protruding from the bilayer. With increasing compressive strain, the morphological response shifted from spherical to tubular protrusions, as monitored by confocal microscopy. In addition, Cambrea et al. showed that spherical protrusions can form on fluid-phase SLBs composed of phosphatidylcholine and phosphatidic acid lipids in response to changing ionic strength conditions, as determined by fluorescence microscopy [130]. The presence of phosphatidic acid (PA) molecules in the SLB caused a large negative spontaneous curvature in the bilayer, leading to deformation of the membrane to induce spherical caps when the SLB was exposed to asymmetric osmotic pressure conditions. It was identified that different geometries of the intercalating lipid molecules significantly affected membrane strain. Following this line, Seu et al. characterized how single-chain lipid amphiphiles affect SLB properties by correlating changes in membrane fluidity with corresponding lipid-phospholipid intermolecular interactions on the basis of fluorescence recovery after photobleaching (FRAP) and attenuated total reflection-fourier transform infrared spectroscopy (ATR-FTIR) experiments [131]. Specifically, it was determined that insertion of a single-chain lipid amphiphile, lysophosphatidylcholine (LPC), in the SLB platform increased membrane fluidity by reducing interactions between phospholipid molecules.

**Figure 6:**
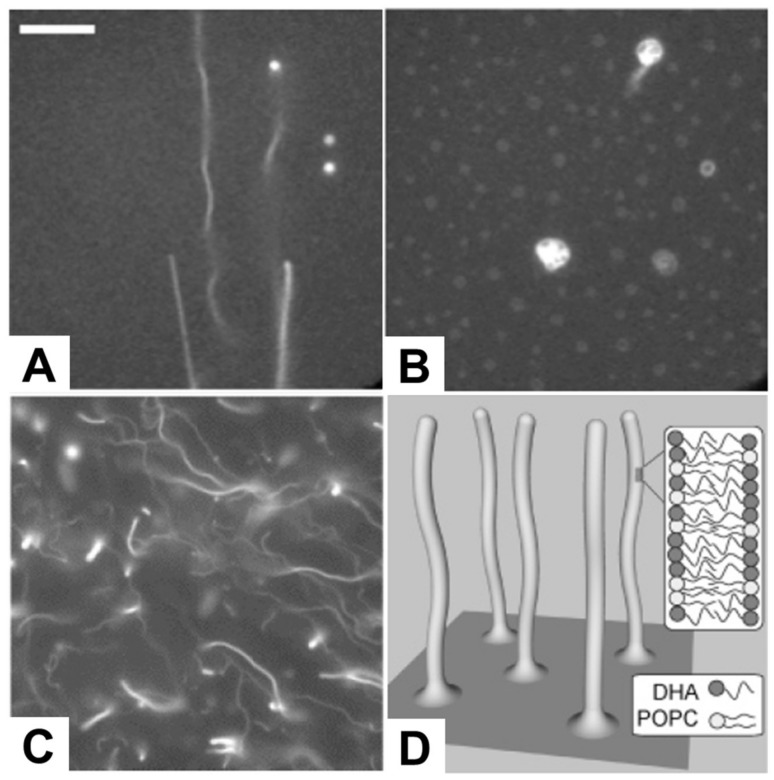
Fluorescence micrographs depicting the morphological responses of SLBs. The morphological responses occurred after treatment with 50 µM lysophosphatidylcholine (LPC) in (**A**) 250 mM KCl and (**B**) 50 mM KCl, and (**C**) 200 µM docosahexaenoic acid (DHA) and corresponding (**D**) proposed mechanism of tubule formation. The scale bar in part A is 25 µm, and is valid for images in parts A and B. The image in part C is 80 µm × 80 µm. Reproduced with permission from [128] (Copyright 2008, American Chemical Society) and [43] (Copyright 2007, American Chemical Society).

Following this line, several studies have been conducted reporting the direct observation of membrane morphological responses upon treating preformed SLB platforms with antimicrobial lipids, including fatty acids and monoglycerides, as summarized in Table 7. Giger et al. treated SLB platforms composed of dioleoylphosphatidylcholine (DOPC) and dioleoylphosphatidic acid (DOPA) phospholipids with C16 LPC, and monitored resulting membrane responses by time-lapsed fluorescence microscopy and FRAP measurements. As presented in Figure 6A,B, elongated tubule structures were observed at or above 50 µM LPC, and a subsequent decrease in the ionic strength conditions transformed the lipid structures from tubule to spherical caps with complex morphologies [128]. Within the specific context of fatty acids, Thid et al. observed similar tubule formation resulting from adding docosahexaenoic acid (DHA), a polyunsaturated long fatty acid, to POPC SLBs [43]. This study was particularly important because it demonstrated the combined use of the quartz crystal microbalance-dissipation (QCM-D) and fluorescence microscopy techniques to study membrane morphological responses in complementary fashion. This study was particularly significant because it was the first investigation reporting the interaction of a fatty acid molecule with an SLB platform and provided initial evidence suggesting that DHA causes tubule formation above its corresponding CMC value. The fluorescence microscopy results provided direct evidence of tubule formation, as evidenced in Figure 6C,D, while the QCM-D measurements provided corroborating data that DHA treatment increased the viscoelastic properties of the SLB platform. The combination of the two measurement techniques led the authors to conclude that the elongated tubules form in response to membrane strain arising from DHA incorporation. Moreover, Flynn et al. conducted a more detailed QCM-D study investigating how DHA treatment affected SLBs composed of POPC alone, POPC and phosphatidylinositol (PI), or POPC and phosphatidylserine (PS), and monitored the corresponding interaction kinetics [44]. At or above 50 µM DHA, there was a large increase in adsorbed mass and dissipation on POPC SLBs, indicating significantly strong interactions to cause perturbation. However, with the introduction of negatively charged PS or PI molecules to the SLB platform, the DHA interaction became attenuated, likely reflecting some degree of electrostatic repulsion between negatively charged SLBs and anionic DHA molecules.

**Figure 7:**
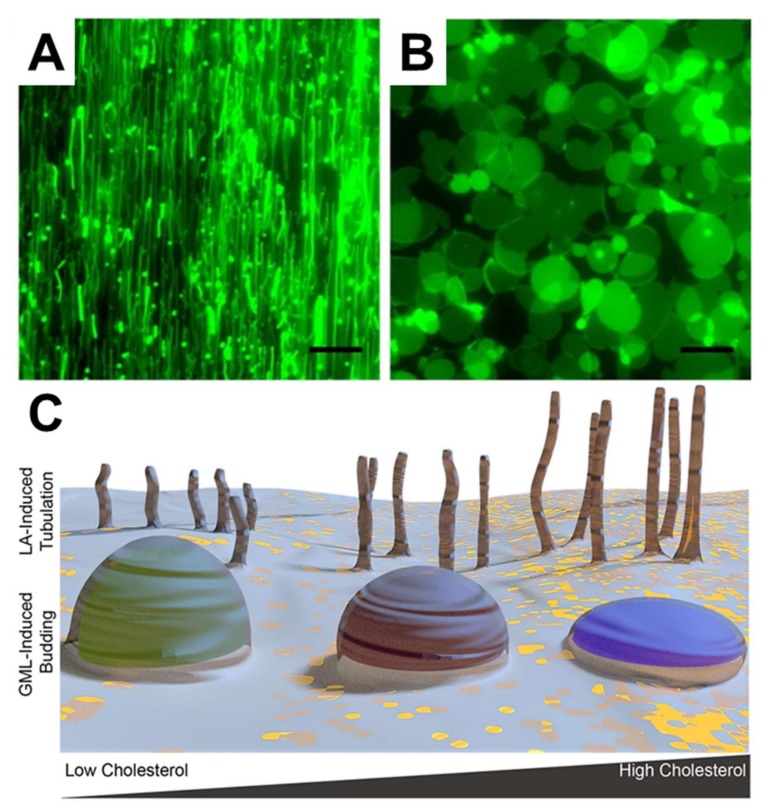
Membrane morphological responses induced by LA and GML. The morphological responses occurred after treating (**A**) 2 mM LA and (**B**) 500 µM GML on DOPC SLBs. The scale bars are 20 μm. (**C**) schematic representation of how membrane morphological responses are induced on cholesterol-rich SLBs with treatment of LA and GML. Reproduced with permission from [109] (Copyright 2015, American Chemical Society) and [133] (Copyright 2017, American Chemical Society).

In terms of more potent and well-known antimicrobial lipids, Yoon et al. employed SLB platforms composed of zwitterionic DOPC phospholipids, to investigate how LA and GML induce membrane morphological responses in SLB platforms [109]. By employing QCM-D and fluorescence microscopy measurements, it was discovered that LA and GML both exhibit membrane-disruptive behavior against SLBs principally above their respective CMC values. Importantly, similar concentration-dependent behavior was observed in the biophysical experiments and biological experiments performed in parallel, specifically MIC determinations against *S. aureus*. However, the two compounds induced strikingly different types of membrane morphological responses, suggesting that antibacterial activity might result from different types of membrane interactions. In particular, LA and GML promoted the formation of elongated tubules and spherical buds formations, respectively, as presented in Figure 7A,B. These findings provide the first evidence that different classes of antimicrobial lipids can interact with phospholipid membranes in distinct ways, and a physicochemical explanation was provided based on how lipid charge (i.e., anionic fatty acids and nonionic monoglycerides) influences membrane translocation rates and corresponding effects on membrane strain. Further investigation of capric acid and monocaprin was conducted using similar experimental strategies as well [132]. Capric acid showed membrane-disruptive activity against SLBs, induced tubule formation, and increased membrane bilayer fluidity only above its CMC value. By contrast, monocaprin was active against SLBs and increase bilayer fluidity both above and below its CMC. Interestingly, monomeric and micellar monocaprin induced different types of membrane morphological response, namely elongated tubules and spherical buds, respectively. Furthermore, Kawakami et al. investigated how the presence of sterols influence membrane morphological responses triggered by antimicrobial lipids [133]. To do so, cholesterol-enriched SLBs were fabricated and it was determined that LA induced tubule formation in all cases, and the extent of membrane remodeling was greater in SLBs with higher cholesterol fractions. In marked contrast, GML addition led to bud formation and the extent of membrane remodeling was lower in SLBs with higher cholesterol fractions, as schematically depicted in Figure 7C. These distinct trends were explained in part by how cholesterol influences the elastic (stiffness) and viscous (stress relaxation) properties of SLB platforms, highlighting the importance of correlating biological activities with detailed biophysical insights.

While the aforementioned studies involved SLB platforms composed of synthetic membrane compositions, Hyldgaard et al. also investigated how monocaprylate, the monoglyceride derivative of caprylic acid, causes membrane destabilization on SLBs composed of bacterial lipid extracts. QCM-D measurements showed a monotonic increase in bound mass with increasing monocaprylate concentration from 0.05 mM upwards, and appreciable shifts were detected at 5 mM test concentration, however, it was difficult to interpret the origin of the measurement responses due to the complex compositional features of the SLB platform in this case. Using AFM, it was further shown that monocaprylate preferentially interacted with liquid-disordered phase regions of the SLB platform and caused membrane defects, suggesting that the membrane interaction of monocaprylate affected membrane fluidity [45]. The studies summarized in this section support the potential of utilizing SLB platforms to study antimicrobial lipids, especially those causing cell lysis, and further translation of these characterization efforts into antimicrobial therapies is a key direction with enormous potential.

## 4. Examples of Therapeutic Applications

Based on the experimental capabilities being developed to study antimicrobial lipids, new insights are being obtained into how these molecules might work therapeutically to treat and prevent bacterial infections. From biological activity profiling, there is detailed information about the relative potency of free forms of antimicrobial lipids, while biophysical experiments are guiding us to better understand how structure-function relationships influence antibacterial activity and can be translated into new classes of self-assembled nanomedicines. Such capabilities are being developed for various systemic and topical applications, and the following examples highlight how antimicrobial lipids are poised to offer safe and effective medicines.

### 4.1. Systemic Treatment of Stomach Infection

*Helicobacter pylori* is a pathogenic Gram-negative bacterium that colonizes the stomach of over half of the world’s population, and is a leading cause of stomach infections, chronic gastritis, gastric ulcers, and stomach cancer [134,135]. Indeed, *H. pylori* infection is a serious health problem because it is estimated that ~75% of all stomach cancer cases worldwide are caused by *H. pylori* infection [136]. As the standard first-line treatment for *H. pylori* infection involves a combination of three of four compounds in total (typically a proton-pump inhibitor and/or bismuth along with two of three antibiotics), however, there are several key challenges faced, including poor patient compliance, history of past antibiotic usage, drug side effects, uncertain eradication rate, and most importantly the presence of antibiotic-resistant *H. pylori* strains [137,138,139]. As such, there is strong motivation to identify new antibacterial agents that work against *H. pylori*, particulary ones that are effective with high potency and have a low risk of drug-resistant strains emerging.

In this regard, fatty acids and monoglycerides are promising agents that have demonstrated antibacterial efficacy against *H. pylori* and there is a high barrier for drug-resistant strains to emerge. The antibacterial activity of fatty acids and monoglycerides was identified through extensive in vitro screening to detremine the antibacterial spectrum of various fatty acids and derivatives thereof. Petschow et al. demonstrated that LA and monoglycerides containing 10–14 carbon long chains effectively killed *H. pylori* at 1 mM treatment concentrations, with a low tendency for resistance development [62]. Similarly, in vitro bacterial susceptibility tests showed that saturated fatty acids and corresponding monoglycerides with 10–14 carbon long chains as well as two additional unsaturated fatty acids and monoglycerides—palmitoleic acid and monopalmitolein—showed an appreciable killing effect against *H. pylori* [64]. Among the findings, monocaprin and GML were the most active against *H. pylori*. Subsequently, Sun et al. reported in vitro inhibitory activity of fatty acids and monoglycerides against *H. pylori* as well [54]. According to the study, LA completely killed *H. pylori* at 1 mM concentration and its monoglyceride derivative, GML, showed similar bactericidal action at lower concentrations around 0.5 mM. Additionally, another unsaturated fatty acid, linolenic acid, also exhibited bactericidal activity at 0.5 mM concentration, in line with the GML potency. Polyunsaturated fatty acids such as docosahexaenoic acid (DHA) also have anti-*H.pylori* activity and been reported to reduce *H.pylori*-associated disease pathogenesis in mouse models [140,141,142,143].

To improve delivery of antimicrobial lipids in highly concentated forms (i.e., to avoid dilution effect), liposomal formulations have been developed that encapsulate the active agents within the liposomal bilayer. The development of liposomal formulations containing intercalated fatty acids are first characterized—usually entailing size, zeta potential, and loading characterization—followed by in vitro efficacy and safety studies, and then in vivo testing. Obonyo et al. developed linolenic acid-loaded liposomes (LipoLLA) and identified that LipoLLA effectively killed both spiral and coccoid forms of *H. pylori* by disrupting the bacterial cell membrane [144]. Compared to free linolenic acid, LipoLLA provided greater killing effect and there was a high barrier to resistance emerging. Jung et al. extended this line of investigation to better understand how LipoLLA interacts with and disrupts the membranes surrounding *H. pylori* cells [145]. Linolenic acid and 18-carbon long analogues were prepared in liposomal formulations, and, out of the tested species, it was confirmed that LipoLLA had the most potent antibacterial activity against *H. pylori* and showed complete killing of the bacterium at 200 μg/mL based on causing an increase in bacterial membrane permeability.

Motivated by these in vitro findings, Thamphiwatana et al. further investigated the therapeutic efficacy of LipoLLA to treat *H. pylori* infection in an in vivo mouse model [146]. After systemic administration of LipoLLA, *H. pylori* burden in the mouse stomach was measured in comparison to treatment with free linolenic acid or conventional triple-therapy involving antibiotics. Treatment with LipoLLA showed superior activity to reduce bacterial burden and the level of *H. pylori*-induced proinflammatory cytokines, indicating that LipoLLA is promising therapeutic agent to treat *H. pylori* infection with high biocompatibility as well. The latter feature was further confirmed recently by Zhong and colleagues by demonstrating that LipoLLA administration induced only minor changes in the gastrointestinal microbiota while conventional triple therapy with three antibiotics caused more dramatic changes in the microbiotica [147].

Following this approach, Seabra et al. reported another promising formulation that involves loading DHA into solid lipid nanoparticles [148]. The nanoparticle enhanced bactericidal activity of DHA against *H. pylori* by enabling delivery of poorly water-soluble DHA and the DHA-loaded nanoparticles were not cytotoxic against human adenocarcinoma cells. Taken together, the results highlight that antimicrobial lipids can be developed into therapeutically viable formulations that can be used for in vivo applications.

### 4.2. Topical Treatment of Skin Infection

Acne vulgaris is the most common skin disorder suffered by teenagers and young adults. The responsible bacterium is *Propionibacterium acnes*, which is a Gram-positive anaerobic rod that thrives inside skin pores, and is an important part of the skin microbiome. Under healthy skin conditions, the skin surface is colonized by beneficial strains of *P. acnes*. In marked contrast, diseased conditions are linked to a higher prevalence of pathogenic strains of *P. acnes*, and therapeutic reduction of *P. acnes* cell counts is associated with improved treatment outcomes.

Towards this goal, free fatty acids are promising antimicrobial candidates because they are naturally found on the skin surface as part of the innate immune system and exogenous addition of antimicrobial lipids can provide a therapeutic effect. While the antibacterial properties of the most potent medium-chain fatty acid, LA, have long been confirmed against a wide range of Gram-positive bacteria, only recently was it demonstrated that LA exhibits inhibitory activity against *P. acnes*. In particular, the antibacterial efficacy and skin cell cytotoxicity of LA was investigated in direct comparison to benzoyl peroxide (BPO), which is a first-choice medication for treating moderate acne [20]. In vitro experimental results showed that LA has a 15-times lower MIC value against *P. acnes* as compared to BPO, and the compound also inhibited *S. aureus* and *Staphylococcus epidermidis* (*S. epidermidis*), which are other types of skin bacteria. Among the tested bacteria, *P. acnes* was the most susceptible bacterium to LA treatment. The demonstrated in vitro efficacy was further encouraged by in vivo safety assessments in mice indicating that intradermal injection or epicutaneous application of therapeutically active concentrations of LA is safe. Ultimately, it was shown that LA treatment via both administration routes led to reduction of *P. acnes* cell counts on infected mouse ears, which in turn mitigated infection-related ear swelling and inflammation. In addition to LA, the inhibitory activity of capric acid against *P. acnes* has also been reported [149]. Capric acid was effective in vitro and in vivo, and reduced ear swelling in mice along with mitigating cytokine and chemokine levels, thereby demonstrating a combination of bactericidal and anti-inflammatory properties. However, it was noted that LA still exhibited the best treatment performance in parallel experiments, reinforcing the importance of establishing clear structure-function relationships to guide selection of antimicrobial lipids.

Regarding delivery methods, liposomal formulations have been explored for LA, in part motivated by the need to typical solubilize antimicrobial lipids in organic solvents such as DMSO that can be skin irritants. To this end, Yang et al. reported the characterization and efficacy testing of LA-loaded liposomes (termed “LipoLA”) with size diameters around 120 nm [150]. Due to their amphipathic properties, LA molecules become encapsulated in the liposomal bilayer and it was shown that complete inhibition of *P. acnes* could be achieved with a 51 μg/mL LA concentration in the LipoLA format. This was an improvement over the 80 μg/mL LA concentration required in free form. To understand the mechanism of antibacterial activity, the authors conducted foster resonance energy transfer (FRET) experiments to study the interaction of LipoLA with bacterial cell membranes and observed fusion and lipid exchange between the two membranes. This was further confirmed by electron microscopy experiments showing that LipoLA treatment causes bacterial cell membrane damage, and in vivo efficacy against *P. acnes* was demonstrated using intradermal injection of LipoLA and topical application of LipoLA gel in a mouse ear infection model [151]. Therapeutic doses of LipoLA across the two administration routes exhibited negligible toxicity, in comparison to BPO and salicylic acid—two medicines that are routinely used to treat mild to moderate acne.

In an alternative strategy, LA was loaded into nano-sized micelles to evaluate its antibacterial efficacy and loading capacity [152]. Poly(caprolactone) poly (ethylene glycol)-poly(caprolactone) (PCL-PEG-PCL) micelles were developed as one promising option. After LA was loaded into the micelles, the average particle size of the PCL-PEG-PCL micelles decreased from around 50–198 nm diameter to 27–89 nm. The antibacterial potency also increased, and the MBC value of LA against *P. acnes* was 80 and 40 μg/mL in the free and micellar forms, respectively. Furthermore, it was possible to tune the payload range based on varying the molecular weight of the polymer chains used in the micelles.

Solid lipid nanoparticles (SLNs) are another option for encapsulating antimicrobial lipids, and have gained attention from the cosmetic and dermatological industries. In SLNs, a solid lipid core encapsulates the active compound and release is achieved when appropriately designed SLNs “melt” upon skin contact, leaving behind the lipid mantle. SLNs have attractive features, including simple and scalable manufacturing, high payload, and biodegradability. Recently, LA has been loaded alone and in combination with retinoic acid into SLN carriers [153]. It was possible to achieve high encapsulation efficiency and inhibit growth of *P. acnes*, *S. aureus* and *S. epidermidis*. In the context of antimicrobial lipids, one drawback of the study was that the SLNs were active against *P. acnes*, both without and with encapsulated LA. Hence, the specific inhibitory effect of LA against *P. acnes* could not be determined, however, only SLNs loaded with LA were active against *S. aureus*. The inclusion of stearylamine, a cationic lipophilic amine, within the SLN composition was cited as an additional contributing factor to explain the inhibitory effect. Further testing of SLNs comprising antimicrobial lipids is warranted to develop optimized SLN versions, and it is noteworthy that SLNs in general are emerging as an industrially acceptable vehicle for skin delivery applications. 

As evidenced by these selected examples, a wide range of delivery formats have been explored for treating bacterial skin infections with antimicrobial lipids. It can also be seen that most efficacy studies involve free fatty acids and the high potency of monoglycerides highlights the importance of continuing to further explore them as candidates to treat bacterial skin infections. Indeed, in addition to treating acne vulgaris, other classes of skin infections such as those caused by *S. aureus* might be treated with antimicrobial lipids in free form [154] or advanced formulations [155,156].

## 5. Conclusions

**Figure 8:**
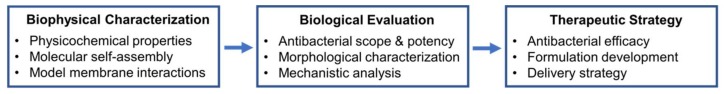
Overview of experimental strategy to characterize antimicrobial lipids based on integrating biophysical and biological approaches.

As presented in this review, there is enormous potential for employing antimicrobial lipids to combat bacterial infections for human health and medicine. Over the past few decades, significant progress has been made towards understanding the relative potency and spectrum of antibacterial activity for different classes of antimicrobial lipids, in turn identifying particularly promising drug candidates through biological investigations. In recent years, these biological investigations have been complemented by biophysical studies aimed at delineating mechanistic properties and correlating membrane interactions with physicochemical parameters such as chain length and headgroup charge. It should be noted that biophysical studies typically involve model systems that are not fully representative of more complex bacterial cell membranes, and hence, extrapolating results to yield biological insights relies on surrogate markers such as profiling molecular-level membrane interactions as an indicator of potential antibacterial activity. Such approaches are particularly useful for studying the molecular basis of interactions involving antimicrobial lipids that cause changes in membrane permeability and induce cell lysis and can predict potency and the potential of synergistic effects. Ultimately, the evolving scope of experimental tools that can be employed to characterize antimicrobial lipids is particularly powerful when integrated into orthogonal measurement strategies that combine biological and biophysical analyses. Figure 8 presents an overview of how we envision that biological and biophysical approaches can be employed synergistically to characterize amphiphilic-based antimicrobial lipids, and to translate this knowledge into biologically relevant therapeutic strategies. Indeed, while conventional drug development focuses on initially screening the biological activity of candidate compounds, we believe that molecular-level characterization can be utilized as a starting point to guide the rational design of therapeutic strategies that take into account molecular design principles. 

By understanding how antimicrobial lipids function and the critical role of molecular self-assembly, we are beginning to design new strategies to enhance therapeutic performance and there is accelerating progress in this direction. Recognizing the challenges of antibiotic-resistant bacteria and taking advantage of the low cost and abundant supply of antimicrobial lipids, there is excellent opportunity to further explore antimicrobial lipids as next-generation antibacterial agents for human health and medicine.

## Figures and Tables

**Table 1:** Antibacterial activity of fatty acids and monoglycerides against different bacteria.

Bacteria (*)	Fatty Acid [FAs]/Monoglycerides [MGs] ^†^	Key Findings	Ref.
*B. megaterium* (+) *B. mycoides* (+) *B. subtilis* (+) *Bacillus* sp. (+) *Strep. faecium* (+) *Strep. lactis* (+) *Staphylococcus* sp. (+) *Micrococcus* sp. (+) *M. lysodeikticus* (+) *Cl. butyricum* (+) *Cl. sporogenes* (+) *Cl. welchii* (+)	[FAs ^‡^]: C8:0, C10:0, C12:0, C14:0, C16:0, C18:0, C18:1, *trans*-C18:1, C18:2, C18:3	Among saturated fatty acids, LA (C12:0) was the most potent against Gram-positive bacteria. Unsaturated fatty acids, linoleic (C18:2) and linolenic acid (C18:3), were more potent than LA.	[50]
Pneumococci (+) Streptococcus group A (+) Streptococcus beta-hemolytic non-A (+) *Corynebacterium* sp. (+) *N. asteroides* (+) *Micrococcus* sp. (+) *S. aureus* (+) *S. epidermidis* (+) Streptococcus group D (+)	[FAs]: C6:0, C8:0, C10:0, C12:0, C14:0, C16:0, C18:0, C14:1, C16:1, C18:1, *trans*-C18:1, C18:2, *trans*-C18:2, C18:3, C20:4 [MGs ^§^]: C10:0, C12:0	LA was the most potent saturated fatty acids against Gram-positive bacteria. Monocaprin (MG C10:0) and GML (MG C12:0) had greater antibacterial activity than fatty acid equivalents. GML had more potent activity with a lower MIC value than LA against most Gram-positive bacteria.	[14]
*Strep. faecalis* (+) *Strep. pyogenes* (+) *S. aureus* (+) *Corynebacterium* sp. (+) *N. asteroides* (+)	[FAs]: C11:0, C12:0, C13:0 [MGs]: C11:0, C12:0, C13:0	LA and GML were the most potent antibacterial compounds among those tested against Gram-positive bacteria. Esterification of fatty acids to monoglyceride form generally increased antibacterial activity.	[15]
*M. smegmatis* (+)	[FAs]: C10:0, C12:0, C14:0, C16:0, C18:0, C20:0, C16:1, C18:1, C18:2, C20:4	Most tested unsaturated fatty acids showed potent bactericidal effect against *M. smegmatis*. Among saturated fatty acids, only LA and myristic acid (C14:0) showed some degree of antibacterial activity at 0.2 mM concentration, which is much weaker than that of unsaturated fatty acids.	[60]
*S. aureus* (+) *L. acidophilus* (+) *B. megaterium* (+) *H. influenzae* (−) *N. gonorrhoeae* (−) *E. coli* (−)	[FA]: C20:4	All tested Gram-positive species were susceptible to treatment with 0.01 mM arachidonic acid (C20:4). *L. acidophilus* was most susceptible among Gram-positive bacteria. Bactericidal effect of arachidonic acid treatment on *S. aureus* depended on treatment time and drug concentration.	[27]
*L. monocytogenes* (+)	[FAs]: C12:0, C14:0, C16:0, C18:0, C18:1, C18:2, C18:3 [MGs] C12:0, C14:0, C16:0	LA, linolenic acid, and GML exhibited strong antibacterial activity against *L. monocytogenes* at 10–20 µg/mL. Bactericidal activity of LA and linolenic acid in brain heart infusion broth was higher at pH 5 than pH 6.	[28]
*B. larvae* (+)	[FAs]: C10:0, C11:0, C12:0, C13:0, C14:1, C16:1, C18:2, etc.	LA and myristoleic acid (C14:1) showed most potent activity against *B. larvae*. Saturated fatty acids with greater than 14- carbon long chains did not inhibit bacterial growth.	[52]
*H. pylori* (−)	[FAs]: C4:0, C5:0, C6:0, C7:0, C8:0, C9:0, C10:0, C11:0, C12:0, C13:0, C14:0, C15:0, C16:0, C17:0, C12:1 [MGs]: C4:0, C5:0, C6:0, C7:0, C8:0, C9:0, C10:0, C11:0, C12:0, C13:0, C14:0, C15:0, C16:0, C17:0, C12:1	Saturated monoglycerides with 10–14 carbon long chains showed bactericidal activity against *H. pylori* with more than 99.99% reduction upon treatment with 1 mM compound. LA was unique among tested saturated medium-chain fatty acids, to have antibacterial activity. Medium-chain monoglycerides inactivated *H. pylori* effectively and there was less spontaneous resistance development.	[62]
*C. trachomatis* (−)	[FAs]: C8:0, C10:0, C12:0, C14:0, C16:1, C18:1 [MGs]: C8:0, C10:0, C12:0, C16:1, C18:1	LA, capric acid (C10:0), and monocaprin had potent antibacterial activity against *C. trachomatis* upon treatment with 10 mM compound. Monocaprin was most potent to kill *C. trachomatis*, as suggested by destabilization of the bacterial membrane of elementary bodies.	[29]
*N. gonorrhoeae* (−)	[FAs]: C8:0, C10:0, C12:0, C14:0, C16:1, C18:1 [MGs]: C8:0, C10:0, C12:0, C14:0, C16:1, C18:1	Monocaprin was the most potent to effectively kill *N. gonorrhoeae*. LA and palmitoleic acid (C16:1) had bactericidal activity against *N. gonorrhoeae*, as determined by treatment with 2.5 mM test compound.	[63]
Streptococcus group A (+) Streptococcus group B (+) *S. aureus* (+)	[FAs]: C8:0, C10:0, C12:0, C14:0, C16:1, C18:1 [MGs]: C8:0, C10:0, C12:0, C14:0, C16:1, C18:1	LA, palmitoleic acid and monocaprin showed strong antibacterial activity against tested *Streptococcus* spp. with 5 mM compound treatment. Monocaprin had significant antibacterial activity against *S. aureus* as well as the *Streptococcus* spp.	[30]
*H. pylori* (−) *E. coli* (−) *Salmonella* spp. (−)	[FAs]: C8:0, C10:0, C12:0, C14:0, C16:1, C18:1 [MGs]: C8:0, C10:0, C12:0, C14:0, C16:1, C18:1	Tested compounds with 10–16 carbon long chains were active against *H. pylori* upon treatment with 10 mM compound, while largely inactive against *Salmonella* spp. and *E. coli*. Monocaprin and GML showed highest levels of inhibitory activity against *H. pylori*.	[64]
*H. pylori* (−)	[FAs]: C4:0, C6:0, C8:0, C10:0, C12:0, C14:0, C16:0, C14:1, C16:1, C18:1, C18:2, C18:3 [MGs]: C12:0, C14:0, C16:0	Among saturated fatty acids, LA was the most potent bactericidal compound against *H. pylori*, with an MBC value of 1 mM. GML was the most potent monoglyceride and had a lower MBC value than LA. Among unsaturated fatty acids, myristoleic and linolenic acid had the most potent antibacterial activity.	[54]
*E. coli* (−)	[FAs]: C2:0, C3:0, C4:0, C5:0, C6:0, C8:0, C10:0, C12:0, C14:0, C16:0, C18:0, C18:1, C18:2	Caprylic (C8:0) and capric acid showed antibacterial activity against *E. coli*; caprylic acid had highest activity. Bactericidal effect of the two fatty acids was higher at pH 5.2.	[65]
*S. enteritidis* (−) *S. infantis* (−) *S. typhimurium* (−)	[FAs]: C2:0, C3:0, C4:0, C5:0, C6:0, C8:0, C10:0, C12:0, C14:0, C16:0, C18:0, C18:1, C18:2	Caprylic acid alone showed antibacterial activity against *Salmonella* species under lower pH conditions around 5.2–5.3.	[66]
*S. aureus* (+) Methicillin-Susceptible *Staphylococcus aureus* (MSSA) (+) Methicillin-Resistant *Staphylococcus aureus* (MRSA) (+)	[FAs]: C8:0, C10:0, C12:0, C14:0, C16:0, C18:0	LA was most potent saturated fatty acids against MSSA and MRSA strains and inhibited their growth at 400 µg/mL test concentration.	[19]
*C. perfringens* (+)	[FAs]: C2:0, C3:0, C4:0, C5:0, C6:0, C8:0, C10:0, C12:0, C14:0, C16:0, C18:0, C18:1, C18:2	LA was the most potent fatty acid against *C. perfringens* and maintained its activity at pH > 6, while capric acid was only active at pH 5.0–5.3.	[31]
*L. garvieae* (+) *V. harveyi* (−) *V. anguillarium* (−) *V. alginocolyticus* (−)	[FAs]: C15:0, C16:0, C17:0, C18:0, C22:0, C18:1, C18:4, C20:4, C20:5, C22:4, C22:5	Unsaturated fatty acids showed greater bactericidal effect on tested bacteria, especially Gram-negative *Vibrio* spp., than saturated fatty acids.	[67]
*B. cereus* (+) *S. aureus* (+) *E. coli* (−) *V. parahaemolyticus* (−) *S. typhimurium* (−) *S. enteritidis* (−)	[FA]: C18:3 [MGs]: C12:0, C14:0	Linolenic acid had the potent antibacterial activity against *B. cereus* and *S. aureus*. Combination of linolenic acid and monoglycerides showed synergistic antibacterial effect compared to treatment with linolenic acid alone.	[68]
*Strep. iniae* (+) *E. ictaluri* (−) *E. tarda* (−) *Y. ruckeri* (−)	[FA]: C8:0 [MG]: C8:0	Antibacterial acitity of caprylic acid and its monoglyceride, monocaprylate ws investgated against bacterial fish pathogens and showed potent efficacy. Monocaprylate showed greater antibacterial acitivty in the 2.5–5 mM range than that of caprylic acid.	[69]
*P. acnes* (+) *S. aureus* (+) *S. epidermidis* (+)	[FA]: C12:0	LA had greater antibacterial activity against *P. acnes* than benzoyl peroxide (BPO). LA was not cytotoxic against human sebocytes.	[20]
*S. aureus* (+) *B. subtilis* (+) *E. coli* (−)	[MG]: C12:0	GML was investigated as a preservative against food-related pathogens. Antibacterial activity of GML was synergisticwith the addition of nisin against tested bacteria.	[70]
*S. aureus* (+) *Strep. pyogenes* (+)	[FA]: C12:0 [MG]: C12:0	GML had 200-fold greater bactericidal potency than LA against *S. aureus*. *Strep. pyogenes* was more susceptible to GML treatment, as compared to *S. aureus*.	[21]
*S. aureus* (+) *B. cereus* (+) *E. coli* (−) *P. aeruginosa* (−)	[MGs]: C11:0, C11:1	MG C11:0 and MG C11:1 effectively inhibited growth of *S. aureus* and *B. cereus*, but were ineffective against tested Gram-negative bacteria.	[71]
*C. sakazakii* (−) *C. malonaticus* (−)	[FAs]: C6:2, C8:0, C10:0, C12:0 [MGs]: C6:2, C8:0, C10:0, C12:0	Caprylic acid and monocaprylate had significant antibacterial acitivty against *Cronobacter* strains. Bactericidal activities of monocaprylate are dependent on compoound concentration and temperature.	[72]

* (+) indicates “Gram-positive bacteria” and (−) indicates “Gram-negative bacteria”; ^†^ [number of carbon atoms in alkyl chain:number of double bonds]; ^‡^ FA indicates “fatty acid”; ^§^ MG indicates “monoglyceride”.

**Table 2:** Summary of experimental approaches to characterize antimicrobial lipids.

	Platform	Technique	Technical Points
**Biological Approaches**	Growth Inhibition Assays	Minimum inhibitory concentration (MIC)	Determines the minimum concentration of antimicrobial lipids that inhibit bacterial growth. Evaluates capacity of a drug candidate by rapid screening of antibacterial activity against target bacteria. Does not provide direct information about the mechanism of antibacterial activity.
	Infectivity Assays	Minimum bactericidal concentration (MBC)	Determines the lowest concentration of antimicrobial lipid to kill a target bacterium. Evaluates if antibacterial activity is bacteriostatic or bactericidal. Does not provide direct information about interaction mechanism.
	Electron Microscopy	Transmission electron microscopy (TEM) Scanning electron microscopy (SEM)	Enables direct observation of antibacterial effects of antimicrobial lipids against target bacterium. Visualizes morphological effects caused by treating target bacterium with antimicrobial lipids. Requires high concentration of antimicrobial lipids (≥2 mM) to treat target bacterium. Bacterial specimen must be fixed and prepared accordingly before imaging; real-time analysis is not possible.
**Biophysical Approaches**	Solution-Phase Liposomes (SUVs and LUVs)	Dynamic light scattering (DLS) Electron microscopy	Monitors interaction kinetics between antimicrobial lipids and phospholipid membranes by measuring changes in the size distribution of liposomes in bulk solution. Can utilize wide range of lipid compositions, including simple ones that are easy-to-prepare. Utilizes DLS as an ensemble-average measurement technique to determine in real-time the size and polydispersity of liposomes in bulk solution, and electron microscopy to visualize how antimicrobial lipids induce morphological changes in individual liposomes post-treatment.
	Giant Unilamellar Vesicle (GUV)	Phase-contrast microscopy Fluorescence microscopy	Visualizes morphological response induced by treatment with antimicrobial lipids in real-time. Can provide deep insights into morphological behaviors, including fluctuations and membrane fission/fusion.
	Supported Lipid Bilayer (SLB)	Quartz crystal microbalance-dissipation (QCM-D) Fluorescence microscopy Fluorescence recovery after photobleaching (FRAP)	Monitors interaction kinetics between antimicrobial lipids and phospholipid membranes. Can be utilized with a wide range of surface-sensitive measurement techniques, allowing detailed investigation of binding mass, change in viscoelastic properties, and membrane fluidity.

**Table 3:** Selected MIC values of fatty acids against different Gram-positive bacteria.

Bacteria	Fatty Acids * (Number of Carbon Atoms in Alkyl Chain:Number of Double Bonds)								Ref.
	C10:0	C12:0	C14:0	C16:0	C18:0	C18:1	C18:2	C18:3	
*B. megaterium*	1.0 mM	0.15 mM	0.15 mM	0.3 mM	0.4 mM	0.05 mM	0.02 mM	0.02 mM	[50]
Pneumococci	1.45 mM	0.062 mM	0.218 mM	0.48 mM	NI ^†^	NI	0.044 mM	0.179 mM	[14]
Streptococcus group A	1.45 mM	0.124 mM	0.547 mM	3.9 mM	NI	1.77 mM	0.089 mM	0.35 mM	
Streptococcus group D	5.8 mM	2.49 mM	4.37 mM	NI	NI	NI	NI	NI	
Streptococcus beta-hemolyticnon-A	2.9 mM	0.249 mM	2.18 mM	3.9 mM	NI	NI	0.089 mM	0.35 mM	
*Micrococcus* sp.	2.9 mM	0.624 mM	0.547 mM	1.9 mM	NI	NI	0.089 mM	0.488 mM	
*Corynebacterium* sp.	1.45 mM	0.124 mM	0.437 mM	1.9 mM	NI	NI	0.044 mM	0.179 mM	
	--	31 µg/mL	--	--	--	--	--	--	[15]
*N. asteroides*	1.45 mM	0.124 mM	0.547 mM	NI	NI	NI	0.089 mM	0.448 mM	[14]
	--	62 µg/mL	--	--	--	--	--	--	[15]
*S. epidermidis*	2.9 mM	2.49 mM	2.18 mM	3.9 mM	NI	NI	NI	NI	[14]
	--	3.9 µg/mL	--	--	--	--	--	--	[20]
*S. aureus*	2.9 mM	2.49 mM	4.37 mM	NI	NI	NI	NI	1.79 mM	[14]
	--	500 µg/mL	--	--	--	--	--	--	[15]
	--	0.97 µg/mL	--	--	--	--	--	--	[20]
MSSA	800 µg/mL	400 µg/mL	1600 µg/mL	>1600 µg/mL	>1600 µg/mL	--	--	--	[19]
MRSA	800 µg/mL	400 µg/mL	1600 µg/mL	>1600 µg/mL	>1600 µg/mL	--	--	--	
*Strep. faecalis*	--	500 µg/mL	--	--	--	--	--	--	[15]
*Strep. pyogenes*	--	62 µg/mL	--	--	--	--	--	--	
*P. acnes*	--	3.9 µg/mL	--	--	--	--	--	--	[20]

* Capric acid (C10:0), LA (C12:0), Myristic acid (C14:0), Palmitic acid (C16:0), Stearic acid (C18:0), Oleic acid (C18:1), Linoleic acid (C18:2), and Linolenic acid (C18:3); ^†^ NI indicates that no bacterial growth inhibition was observed within the tested concentration range.

**Table 4:** Selected MIC values of monoglycerides against different Gram-positive bacteria.

Bacteria	Monoglycerides * (Number of Carbon Atoms in Alkyl Chain:Number of Double Bonds)			Ref.
	C10:0	C12:0	C13:0	
Pneumococci	0.1 mM	0.09 mM	--	[14]
Streptococcus group A	0.2 mM	0.045 mM	--	
Streptococcus group D	2.0 mM	NI ^†^	--	
Streptococcus beta-hemolyticnon-A	0.2 mM	0.09 mM		
*Micrococcus* sp.	0.1 mM	0.09 mM	--	
*S. epidermidis*	1.0 mM	0.09 mM	--	
*Corynebacterium* sp.	0.2 mM	0.045 mM	--	
	--	16 µg/mL	NI	[15]
*N. asteroides*	0.5 mM	0.09 mM	--	[14]
	--	16 µg/mL	125 µg/mL	[15]
*S. aureus*	1.0 mM	0.09 mM	--	[14]
	--	250 µg/mL	NI	[15]
*Strep. faecalis*	--	NI	NI	
*Strep. pyogenes*	--	8 µg/mL	62 µg/mL	

* Glycerol monocaprate (monocaprin) (C10:0), GML (C12:0), glycerol monomyristate (C14:0); ^†^ NI indicates that no bacterial growth inhibition was observed within the tested concentration range.

**Table 5:** Electron microscopy studies reporting how antibacterial fatty acids and monoglycerides affect bacterial cells.

Antimicrobial Lipid	Bacteria *	Technique ^†^	Key Observations	Ref.
Oleic acid	Streptococcus group A (+)	TEM	Oleic acid aggregates around individual cells of group A streptococci and interacts with the bacterial cell membrane. Cytoplasmic changes occurred upon treatment, inducing vacuolization and nucleoid aggregation.	[26]
Arachidonic acid	*N. gonorrhoeae* (−) *S. aureus* (+)	TEM	Disruption of *N. gonorrhoeae* bacterial membrane was induced by 10 µM arachidonic acid treatment, leading to morphological deformation and leakage of cytoplasmic contents. Similar treatment of *S. aureus* cells did not cause significant change of the cell wall structure, while condensation of cytoplasmic contents and other effects were observed.	[27]
Linolenic acid Glycerol Monolaurate (GML)	*L. monocytogenes* (+)	TEM	Significant lysis of *L. monocytogenes* cells was observed upon treatment with 50 µg/mL GML. Leakage of cytoplasmic contents was detected from GML-treated cells. Upon treatment with linolenic acid, cells exhibited irregular surface morphologies without apparent lysis.	[28]
Monocaprin	*C. trachomatis* (−)	TEM	*C. trachomatis* in elementary body form exhibited morphological deformations and shrunken shape upon treatment with 10 mM monocaprin.	[29]
Monocaprin	Streptococcus group B (+)	TEM SEM	Upon treatment with 10 mM monocaprin, the surface morphology of Streptococcus group B appeared to be unaffected, as determined by SEM. Disruption of plasma cell membrane and disappearance of granules were observed by TEM.	[30]
Lauric acid (LA)	*C. perfringens* (+)	TEM	Upon treatment with 1 mg/mL LA, complete cell membrane separation and disruption of *C. perfringens* cells were observed, but there was no structural change in the cell wall. Cytoplasmic contents became disordered upon treatment.	[31]
Eicosapentaenoic acid (EPA)	*S. aureus* (+) *P. aeruginosa* (−)	SEM	Upon treatment with EPA, severe morphological deformations along with the appearance of irregular bacterial surfaces were observed. Upon treatment, *S. aureus* cells lost regular shape and became rough, while hollow structures became evident on the surface of *P. aeruginosa* cells.	[32]

* (+) indicates “Gram-positive bacteria” and (−) indicates “Gram-negative bacteria”; ^†^ TEM and SEM indicate transmission electron microscopy and scanning electron microscopy, respectively.

**Table 6:** Investigation of fatty acid interactions with solution-phase liposomes.

Liposome (Composition)	Fatty Acid/Anion	Techniques	Key Observations	Ref.
SUVs (POPC)	Oleate	Electron microscopy Dynamic light scattering UV/VIS spectrophotometry	Different molar ratio of oleate were added to preexisting POPC liposomes, and caused varying changes in the size and number of newly formed mixed liposomes. At a low molar ratio of oleate to POPC liposomes, the size of resulting mixed liposomes remained similar, while at high ratios, the mixed liposomes were larger and became polydisperse.	[33]
SUVs (POPC)	Oleate	Cryo-TEM UV/VIS spectrophotometry	Oleate addition to preexisting POPC liposomes induced rapid formation of POPC/oleate mixed liposomes with increased diameter and total number. A few smaller mixed liposomes were generated by induced fission processes.	[34]
SUVs (POPC)	Oleate	Cryo-TEM	Equimolar addition of oleate to preexisting POPC liposomes led to smaller size distributions of new mixed liposomes, and occurred via subsequent fission processes.	[35]
SUVs (POPC)	Oleate	Dynamic light scattering Optical density	Addition of oleate to preexisting POPC SUVs (50, 100 nm) induced formation of mixed POPC/oleate liposomes with similar size distributions to original ones.	[36]
SUVs, LUVs (Egg PC)	Oleate	Gel filtration chromatography combined with dynamic light scattering (DLS) UV/VIS spectrophotometry	In the presence of Egg PC vesicles, oleate induced rapid spontaneous vesiculation, forming new EggPC/oleate vesicles. Size distribution of the formed EggPC/oleate vesicles depended on preexisting Egg PC vesicle size.	[37]
SUVs, LUVs (Egg PC)	Oleate	Electron microscopy Dynamic light scattering Gel exclusion chromatography	Size distribution of mixed phospholipid/oleate liposomes depended on the amount of oleate added to preexisting phospholipid liposomes. Small amount of added oleate induced a narrower size distribution of the mixed liposomes, as compared to when a larger amount was added.	[38]
SUVs, LUVs (Egg PC)	Oleate	Gel exclusion chromatography	Addition of oleate to preexisting liposomes induced the formation of smaller mixed phospholipid/oleate liposomes. Two different mechanisms, fission and partial solubilization, were attributed to causing formation of smaller vesicles.	[39]
SUVs (DMPC, POPC)	Capric acid, Oleic acid, Linoleic acid	Electron microscopy Dynamic light scattering Light microscopy UV/VIS spectrophotometry	Rapid incorporation of fatty acids into preexisting liposomes induced formation of mixed phospholipid/fatty acid liposomes through size growth and subsequent fission. Fatty acids were more likely to incorporate into the preexisting liposomes than forming fatty acid vesicles themselves.	[40]

**Table 7:** Interactions of antimicrobial lipids and related single-chain lipid amphiphiles with SLB platforms.

SLB Composition	Single-Chain Amphiphiles	Techniques	Key Observations	Ref.
DOPC/PA	Lysophosphatidylcholine (LPC)	Fluorescence microscopy FRAP	At or above 50 µM LPC concentration, elongated tubule protrusions formed from SLB. Decrease in ionic strength shifted membrane structure from tubule to spherical cap shape.	[128]
Egg PC	LPC Lysophosphatidylethanolamine (LPE)	FRAP ATR-FTIR	Addition of LPC increases bilayer fluidity, while addition of LPE decreased bilayer fluidity. Hydrogen bonding interactions between phosphate group of lipids and amine group (in PE headgroup) were cited as cause of decreased fluidity.	[131]
POPC	Docosahexaenoic acid (DHA)	QCM-D Fluorescence microscopy	Above CMC value of DHA (~60 µM), DHA induced significant changes in the viscoelastic properties of the SLB platform. Treatment with 200 µM DHA caused formation of elongated worm-like lipid (tubule) structures.	[43]
POPC, POPC/PS, POPC/PI	Docosahexaenoic acid (DHA)	QCM-D	Effects on POPC SLB induced by DHA treatment were concentration-dependent and occurred at or above 50 µM DHA. Incorporation of PS and PI into POPC SLBs decreased the interaction of DHA with the lipid bilayer.	[44]
DOPC	LA GML SDS	QCM-D Fluorescence microscopy	Significant membrane disruption primarily occurred above the CMC value of each compound. LA and SDS induced elongated tubule formation, while GML induced bud formation.	[109]
DOPC	Capric acid Monocaprin	QCM-D Fluorescence microscopy FRAP	Capric acid disrupted DOPC SLBs only above its CMC value of 3.5 mM. Monocaprin was active against DOPC SLBs both above and below its CMC value, and induced different types of membrane morphological responses above and below CMC.	[132]
DOPC/Cholesterol	LA GML SDS	QCMD	Similar types of membrane morphological responses were observed in cholesterol-free and cholesterol-enriched SLBs. With increasing cholesterol fraction, LA and SDS induced greater membrane remodeling, while GML effect became smaller.	[133]
Bacterial lipid extracts (*E. coli*)	Monocaprylate	QCM-D AFM	At or above 5 mM monocaprylate concentrations, significant changes in the viscoelastic properties of *E. coli* lipid SLBs occurred. It was suggested that monocaprylate interacts with SLBs in the liquid-disordered phase state and causes defect formation.	[45]
